# Inhibitory proteins of *Bacillus subtilis* interact within the membrane to block intramembrane protease activity

**DOI:** 10.1128/jb.00186-25

**Published:** 2025-10-08

**Authors:** Saikat Mandal, Alanah Soriano, Caroline Erpelding, Jackson Ruffner, Eric Smith, Benjamin J. Orlando, Lee Kroos

**Affiliations:** 1 Department of Biochemistry and Molecular Biology, Michigan State University 3078, East Lansing, Michigan, USA; University of Massachusetts Chan Medical School, Worcester, Massachusetts, USA

**Keywords:** BofA, SpoIVFA, SpoIVFB, sporulation, *Bacillus subtilis*, regulated intramembrane proteolysis, intramembrane protease

## Abstract

Intramembrane proteases (IPs) are crucial for diverse signaling pathways, including some that regulate the virulence of pathogenic bacteria. A better understanding of mechanisms controlling IP activity is necessary to guide therapeutic development. *Bacillus subtilis* SpoIVFB is an IP with two natural inhibitory proteins, BofA and SpoIVFA. These proteins form a complex with SpoIVFB and prevent it from cleaving Pro-σ^K^ during endosporulation. We investigated proximity between BofA and SpoIVFA in the SpoIVFB inhibition complex using *in vivo* disulfide crosslinking in *Escherichia coli*. We discovered that two parts of the BofA C-terminal region are proximal to the SpoIVFA transmembrane segment (TMS). Our results support predictions that the BofA C-terminal region adopts an unusual structure within the membrane and interacts with the SpoIVFA TMS to block SpoIVFB cleavage of Pro-σ^K^. Endospore-forming *Bacilli*, including several pathogenic species, encode BofA and SpoIVFA orthologs, which likely interact in a similar fashion to inhibit their SpoIVFB ortholog. It may be possible to control SpoIVFB activity and hence endosporulation with modulators that target the interaction between BofA and SpoIVFA.

**IMPORTANCE:**

Regulated intramembrane proteolysis (RIP) pathways govern important processes in all three domains of life. A key component of RIP pathways is an intramembrane protease (IP), which cleaves one or more substrates within a membrane. Developing modulators of IPs has been challenging, particularly for metallo-IPs. Bacterial metallo-IPs function in RIP pathways that control the virulence of many pathogens. SpoIVFB is a metallo-IP necessary for endosporulation of *Bacillus subtilis*. Endosporulation enhances the survival of pathogenic *Bacilli*, which encode orthologs of SpoIVFB and its natural inhibitory proteins BofA and SpoIVFA. Here, we present the first experimental evidence for contacts between BofA and SpoIVFA within the membrane-embedded SpoIVFB inhibition complex, providing foundations for a deeper understanding of mechanisms controlling metallo-IP activity.

## INTRODUCTION

Regulated intramembrane proteolysis (RIP) controls diverse biological processes in all three domains of life (1, 2). RIP typically involves one or more initial cleavages by a soluble protease(s) or a soluble domain of a membrane-associated protease (3, 4). The initial cleavages may target a regulatory protein(s) or the eventual substrate(s) of an intramembrane protease (IP). The initial cleavages enable the substrate(s) to access the membrane-embedded IP active site (5–8). The IP cleaves the substrate(s) within the membrane, releasing substrate fragments from the membrane, and these soluble protein fragments control one or more cellular functions (9, 10). For example, human site-2 protease (S2P) is a metallo-IP that cleaves membrane-associated transcription factors, governing cholesterol homeostasis, the endoplasmic reticulum stress response, and viral infection (1, 11, 12). In bacteria, S2P-related metallo-IPs control stress responses, pathogenicity, and other functions (4, 13–16).

A deeper understanding of RIP pathways is necessary to enable modulation of processes that impact human health and welfare. Over the past 25 years, tremendous progress has been made toward understanding the structure and function of IPs, including in some cases their substrates and/or inhibitors (2, 17–23). Increasingly, new insights with direct therapeutic potential are emerging (16, 24–27).

**Fig 1:**
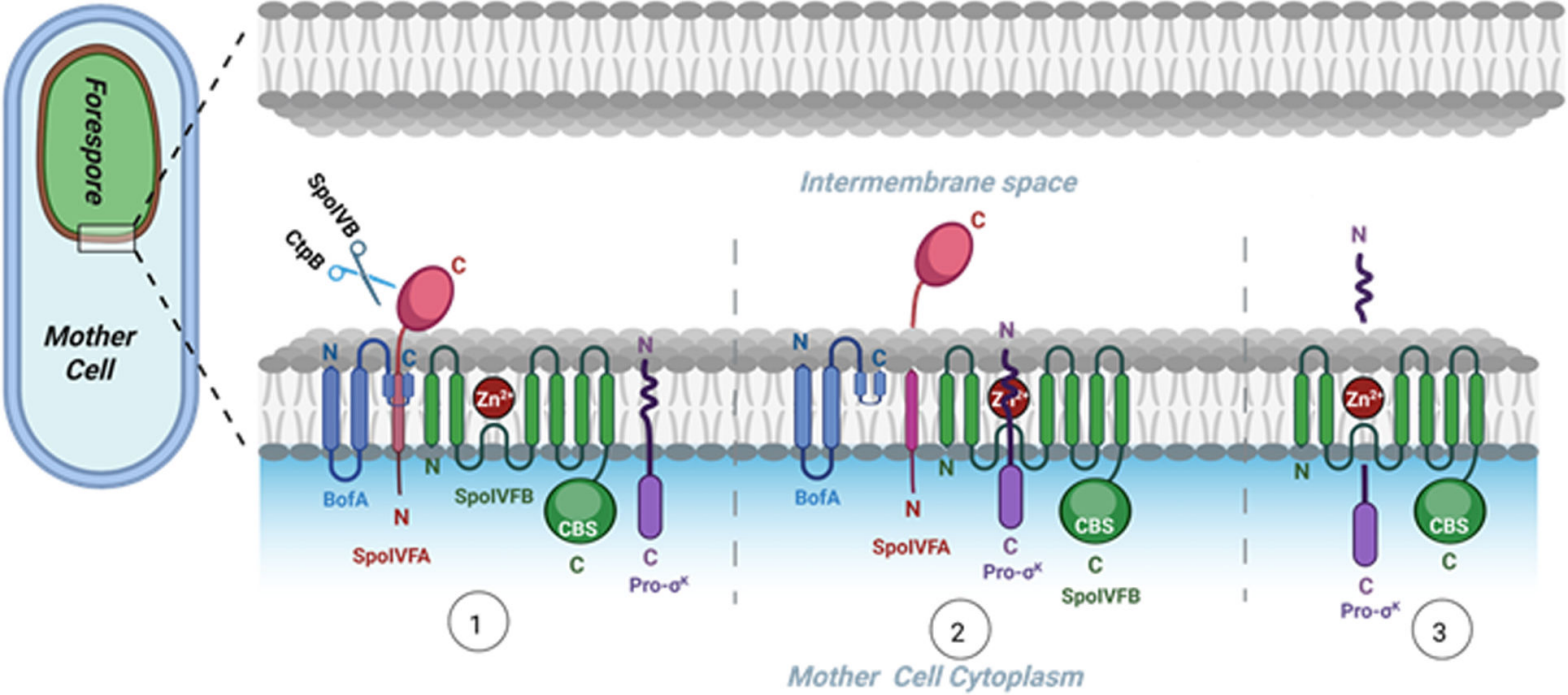
Regulated intramembrane proteolysis (RIP) of Pro-σ^K^ in sporulating *Bacillus subtilis*. At left is a diagram of a sporulating cell. At right is a closer view of the two membranes surrounding the forespore and the steps involved in RIP of Pro-σ^K^. In step 1, BofA and SpoIVFA prevent SpoIVFB from cleaving Pro-σ^K^ until proteases SpoIVB and CtpB are secreted from the forespore and cleave the SpoIVFA C-terminal domain. In step 2, the N-terminal Proregion of Pro-σ^K^ enters the SpoIVFB active site region, which includes a zinc ion. In step 3, the zinc ion and SpoIVFB residues catalyze Pro-σ^K^ cleavage, releasing σ^K^ into the mother cell cytoplasm to direct transcription.

A RIP pathway controlling cleavage of the membrane-associated transcription factor Pro-σ^K^ by the S2P-related metallo-IP SpoIVFB involves signaling between the forespore and mother cell compartments during endospore formation (Fig. 1). This process is well-characterized in *Bacillus subtilis* (13, 28); however, gaps remain in our knowledge of how SpoIVFB activity is regulated. BofA and SpoIVFA inhibit SpoIVFB activity late in endosporulation (29–31) (Fig. 1, step 1), but the mechanism of inhibition is unclear. Experiments with sporulating *B. subtilis* suggest that BofA and SpoIVFA prevent Pro-σ^K^ association with SpoIVFB (32). However, Pro-σ^K^ appeared to be associated with SpoIVFB, BofA, and SpoIVFA in *Escherichia coli* engineered to overproduce all four proteins, and the inhibitory proteins appeared to block substrate access to the SpoIVFB active site (8).

**Fig 2:**
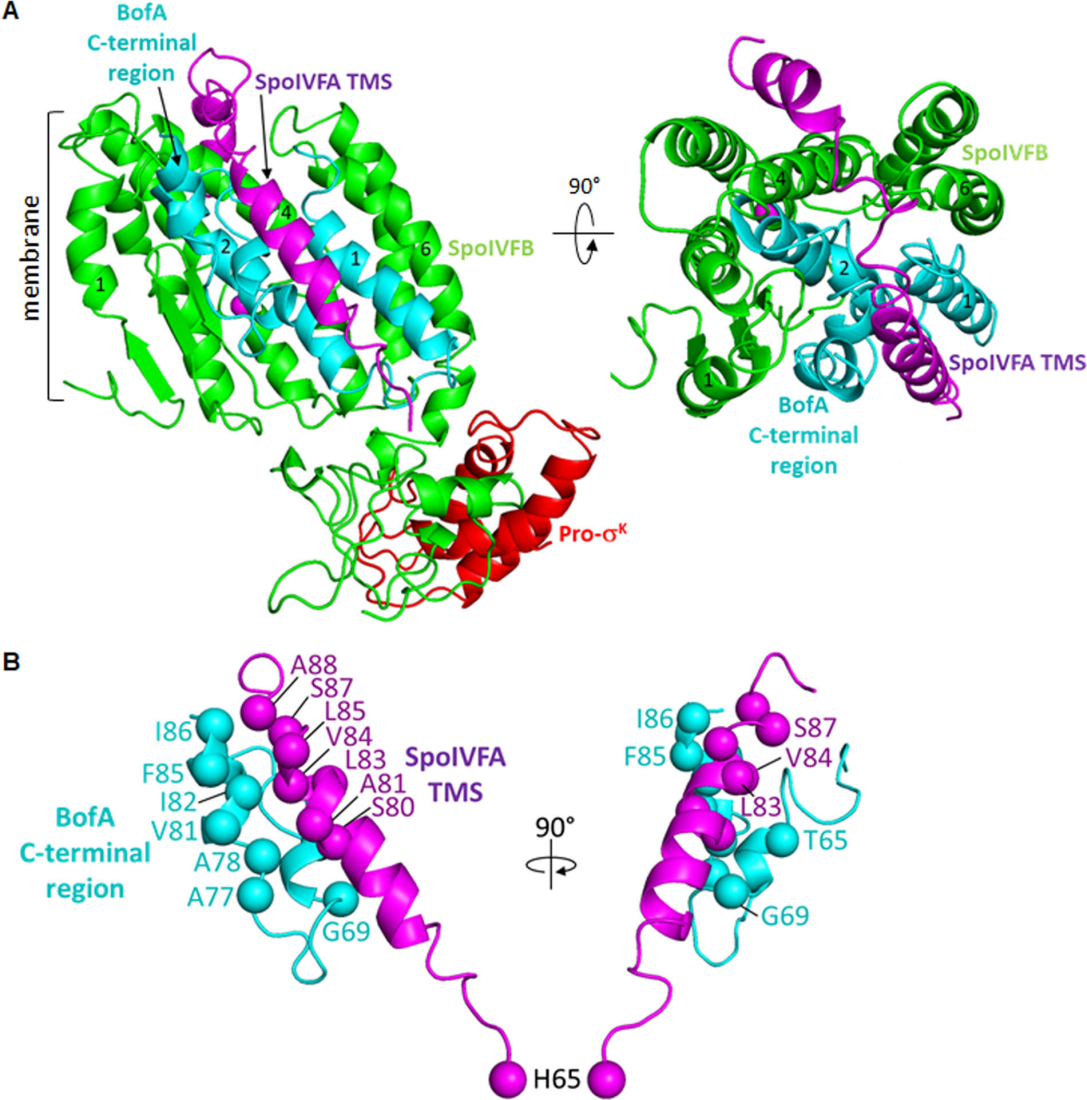
Model of the SpoIVFB inhibition complex. (**A**) SpoIVFB with BofA and parts of SpoIVFA and Pro-σ^K^. At left is a side view with BofA transmembrane segment 2 (TMS2) in the SpoIVFB active site cleft between TMS1 and TMS6. At right is a top view showing only the SpoIVFB membrane domain with BofA and SpoIVFA residues 65-111. The SpoIVFA TMS is predicted to be near the BofA C-terminal region. SpoIVFA is also predicted to interact with SpoIVFB TMS4. (**B**) Predicted interaction between parts of the inhibitory proteins. The BofA C-terminal region (residues 55-87) is predicted to be proximal to the SpoIVFA TMS (residues 72–92). SpoIVFA H65 is predicted to be distal from the BofA C-terminal region. At left is a side view as in panel A, with the C_α_ atoms of residues tested for disulfide crosslinking shown as spheres. At right is a rotated side view so that BofA T65 can be seen.

Earlier studies showed that primarily SpoIVB and secondarily CtpB, both secreted from the forespore into the intermembrane space, cleave SpoIVFA (33–38), and CtpB may also cleave BofA (39), allowing the Proregion of Pro-σ^K^ to access the SpoIVFB active site (Fig. 1, step 2), but the mechanism of active site access remains a mystery. Ramirez-Guadiana et al. (32) proposed that SpoIVB-dependent cleavage of SpoIVFA triggers a conformational change in SpoIVFB that allows stable association of Pro-σ^K^ and opens a gate between SpoIVFB transmembrane segments 1 and 6 (TMS1 and TMS6) through which the Proregion passes to access the active site. Olenic et al. (8) reported that BofA TMS2 occupies the SpoIVFB active site cleft, blocking access of the Pro-σ^K^ Proregion. A structural model was proposed in which SpoIVFA bridges the BofA C-terminal region and SpoIVFB TMS4, stabilizing the inhibition complex (Fig. 2A). The model implied that relief of SpoIVFB inhibition would require removal or substantial movement of SpoIVFA and BofA (8), presumably due to cleavages of SpoIVFA by SpoIVB and CtpB (33, 34, 36, 37) and possibly the cleavage of BofA by CtpB (39).

Despite uncertainty about how the Pro-σ^K^ Proregion is initially prevented from gaining access to the SpoIVFB active site and how it subsequently gains access, it is clear that the Proregion is cleaved from Pro-σ^K^ by SpoIVFB metalloprotease activity (40–42), releasing σ^K^ into the mother cell cytoplasm (Fig. 1, step 3). This allows formation of σ^K^ RNA polymerase, which transcribes genes for completion of endospore formation (43, 44). Recently, deeper insight into the mechanism SpoIVFB uses to engage its substrate for cleavage emerged (22). The cryo-EM structure of SpoIVFB bound to Pro-σ^K^, and mutational analysis, showed that a membrane reentrant loop of SpoIVFB captures the Proregion in an extended β-sheet conformation to facilitate peptide bond hydrolysis. This β-sheet augmentation mechanism of substrate engagement is conserved broadly across different types of IPs (19, 21–23, 45–47). The cryo-EM structure and disulfide crosslinking in *E. coli* showed that Pro-σ^K^ interacts with the SpoIVFB interdomain linker, which connects TMS6 of its membrane domain to its soluble cystathionine-β-synthase (CBS) domain (22).

Here, we report testing structural models of the SpoIVFB inhibition complex. We focus on the predicted interaction between the two inhibitory proteins (8) (Fig. 2B). The BofA C-terminal region was predicted to adopt an unusual structure within the membrane, consisting of two short α-helices connected by a turn, and this structure was predicted to interact with the TMS of SpoIVFA. We tested this prediction using *in vivo* disulfide crosslinking in *E. coli*, a method in which coexpression of two proteins with a single Cys residue results in disulfide bond formation upon treating the cells with oxidant if the proteins interact and the Cys residues are close together and favorably oriented (48). Cross-linked proteins can be detected by immunoblot. Our results provide evidence that the BofA C-terminal region and the SpoIVFA TMS interact within the membrane to block SpoIVFB activity.

## RESULTS

### The BofA C-terminal region is proximal to the SpoIVFA transmembrane segment in the SpoIVFB inhibition complex

A structural model of the SpoIVFB inhibition complex predicted that the BofA C-terminal region is proximal to the SpoIVFA TMS (8) (Fig. 2B). We refer to this model as the “previous model,” which we used to choose combinations of BofA and SpoIVFA residues that might be close enough to form a disulfide crosslink when substituted with Cys. The combinations we chose to test are shown in Table S1 along with the distance between C_β_ atoms predicted by the previous model (8). Some of the C_β_ distances exceed the 5.4 Å length of a typical C_β_−S−S−C_β_ disulfide bond, but we nevertheless tested these combinations since the model is uncertain and proteins undergo conformational fluctuations; hence, we might observe crosslinking. Conversely, C_β_ distances < 5.4 Å may or may not result in crosslinking, owing to model uncertainty and/or unfavorable side chain orientations. As a negative control, we included the combination of SpoIVFA H65 and BofA T65 with a predicted C_β_ distance of 29.7 Å (Table S1), which we expected to preclude crosslinking.

To test for disulfide crosslinking between the chosen combinations of residues, we coexpressed single-Cys variants of MBPΔ27BofA and SpoIVFA with Cys-less variants of SpoIVFB and Pro-σ^K^(1–127) in *E. coli*. MBPΔ27BofA has the *E. coli* maltose-binding protein (MBP) fused to a variant of BofA lacking its N-terminal 27 residues, which are dispensable for function (8, 49). The MBP provides a tag for immunoblot detection. Likewise, the Cys-less variants of SpoIVFB and Pro-σ^K^(1-127) are tagged with FLAG_2_ and/or His_6_ for immunoblot detection, and a Cys-less variant of functional SpoIVFB can cleave Cys-less Pro-σ^K^(1-127) (50). We transformed derivatives of pET Quartet, a plasmid engineered to coexpress all four proteins, into *E. coli*, where the proteins form a complex in the inner membrane (8). We treated the cells with the oxidant Cu^2+^(phenanthroline)_3_ (Cu) to promote disulfide crosslinking. As controls, we treated cells with the reductant DTT to prevent crosslinking or with Cu followed by DTT to reverse crosslinking.

**Fig 3:**
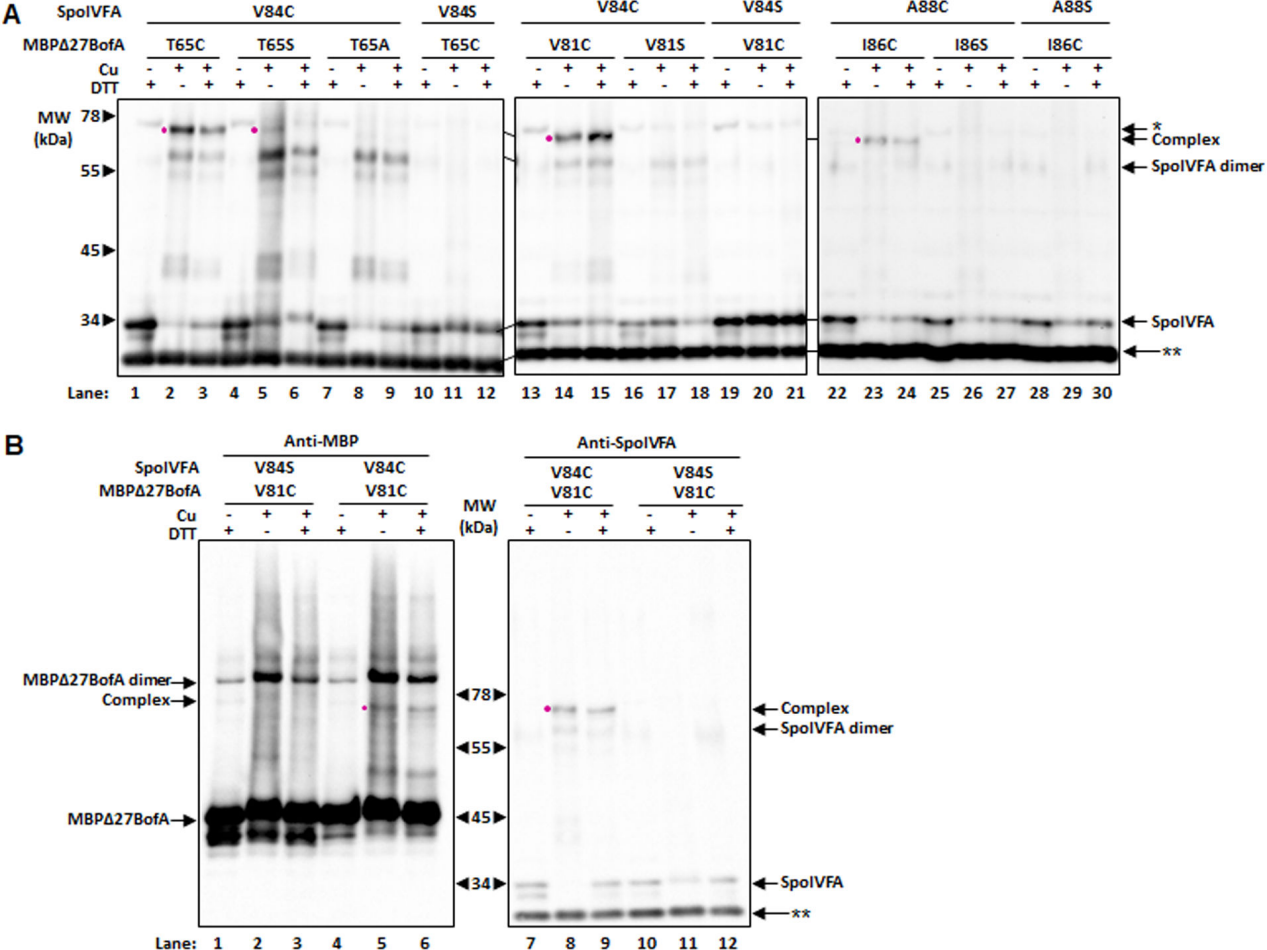
The BofA C-terminal region is close to the SpoIVFA transmembrane segment (TMS). (**A**) Anti-SpoIVFA immunoblots of disulfide crosslinking between SpoIVFA and MBPΔ27BofA variants. The variants lack cysteine residues except at the indicated position. As negative controls, the single cysteine residue was replaced by serine or alanine. Variants were coexpressed in *E. coli*, and the cells were treated with oxidant Cu^2+^(phenanthroline)_3_ (Cu) (1 mM) for 1 h to promote disulfide crosslinking, reductant DTT (100 mM) to inhibit crosslinking, or with Cu followed by DTT to reverse crosslinking. Species migrating at positions expected for SpoIVFA, SpoIVFA dimer, and complex between SpoIVFA and MBPΔ27BofA are indicated. The complex, if present, is also identified with a dot (•) next to the sample treated only with Cu. A star (*) denotes an unidentified species migrating more slowly than complex and most obvious in samples treated only with DTT. A double star (**) indicates a cross-reacting protein. The position of migration of protein molecular weight (MW) markers is shown on the blot at left. Migration was slightly greater on that gel, so lines between the blots indicate their alignment. Representative results from at least two biological replicates are shown. (**B**) Anti-MBP and anti-SpoIVFA immunoblots of the same set of samples subjected to disulfide crosslinking between SpoIVFA and MBPΔ27BofA variants.

We observed disulfide crosslinking for several combinations of single-Cys variants (Fig. 3A). For example, Cu treatment of *E. coli* producing V84C SpoIVFA (with a single-Cys in its predicted TMS) and T65C MBPΔ27BofA (with a single-Cys in its C-terminal region) showed a species migrating at ~75 kDa upon immunoblotting with anti-SpoIVFA antibodies (lane 2). The predicted molecular weight of a crosslinked complex between SpoIVFA (29.6 kDa) and MBPΔ27BofA (46.6 kDa) is 76.2 kDa. Treatment with Cu followed by DTT diminished the intensity of the ~75 kDa species, consistent with partial reversal of a disulfide crosslink (lane 3). The ~75 kDa species was barely visible when we coexpressed V84C SpoIVFA with T65S MBPΔ27BofA as a negative control (lane 5). Although T65S could not form a disulfide crosslink with V84C, we reasoned that perhaps the Ser hydroxyl group could crosslink weakly with the Cys sulfhydryl group; hence, we tested T65A MBPΔ27BofA and did not observe the ~75 kDa species (lane 8). As another negative control, we coexpressed V84S SpoIVFA with T65C MBPΔ27BofA, and as expected, we did not observe the ~75 kDa species (lane 11). Neither did we observe a species migrating at ~60 kDa, the predicted molecular weight of a crosslinked SpoIVFA dimer, which we did observe when we expressed V84C SpoIVFA and treated it with Cu (lanes 2, 3, 5, 6, 8, and 9). Collectively, these results provide evidence that the ~75 kDa species is V84C SpoIVFA crosslinked to T65C MBPΔ27BofA and that the BofA C-terminal region is proximal to the SpoIVFA TMS in the SpoIVFB inhibition complex (Fig. 2B). Importantly, the Cys or Ser substitutions at positions V84 of SpoIVFA or T65 of MBPΔ27BofA did not interfere with inhibition of SpoIVFB (Fig. S1, lanes 7, 8, 11, and 12), showing that these protein variants are functional.

We obtained similar results that support proximity between a second part of the BofA C-terminal region and the SpoIVFA TMS in the SpoIVFB inhibition complex. Cu treatment of *E. coli* producing V84C SpoIVFA and V81C MBPΔ27BofA (with a single-Cys near its C-terminal I87 residue) exhibited the ~75 kDa species expected for crosslinked complex (Fig. 3A, lane 14). Surprisingly, treatment with Cu followed by DTT did not reverse this crosslink (lane 15). On the other hand, as expected, neither V81S MBPΔ27BofA (lane 17) nor V84S SpoIVFA (lane 20) formed the crosslinked complex. Likewise, Cu treatment of *E. coli* producing A88C SpoIVFA and I86C MBPΔ27BofA formed the apparent crosslinked complex (lane 23), unless Ser rather than Cys was substituted for I86 in MBPΔ27BofA (lane 26) or A88 in SpoIVFA (lane 29). The Cys or Ser substitutions at position A88 of SpoIVFA, or either V81 or I86 of MBPΔ27BofA, did not interfere with inhibition of SpoIVFB (Fig. S1, lanes 3–6, 9, 10), showing that these protein variants are functional. Taken together, these results provide evidence that V81 and I86 near the C-terminal end of BofA are proximal to V84 and A88, respectively, near the C-terminal end of the predicted SpoIVFA TMS (Fig. 2B; Table S1).

To determine whether MBPΔ27BofA was present in one of the apparent crosslinked complexes, we performed an experiment in which we subjected two sets of the same samples to immunoblot analysis with anti-MBP or anti-SpoIVFA antibodies. We loaded prestained molecular weight markers in the center lane and a set of samples on either side for SDS-PAGE. After transfer, we cut the blot at the center of the prestained markers and incubated each half with different antibodies. We observed the ~75 kDa species in the expected lanes of both immunoblots (Fig. 3B, lanes 5 and 8), providing additional evidence that the ~75 kDa species is the crosslinked complex, in this case, of V84C SpoIVFA with V81C MBPΔ27BofA. We also observed a species migrating at ~93 kDa, the predicted molecular weight of a crosslinked MBPΔ27BofA dimer, in the immunoblot with anti-MBP antibodies (Fig. 3B, lanes 1–6).

**Fig 4:**
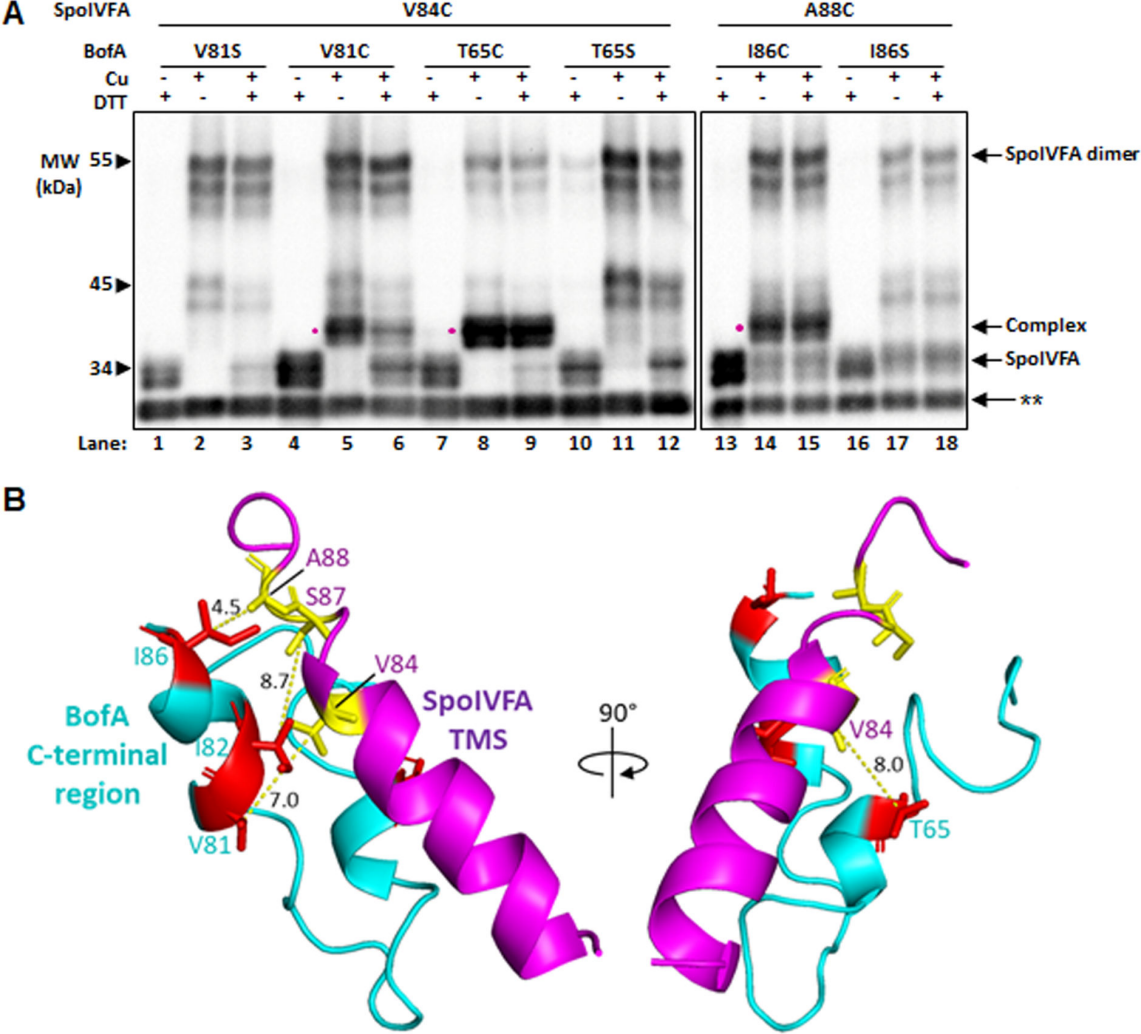
Disulfide crosslinking between variants of full-length BofA and SpoIVFA. (**A**) Anti-SpoIVFA immunoblot. Crosslinking was performed as described in the Fig. 3 legend, except with *E. coli* coexpressing the indicated variants of full-length BofA rather than MBPΔ27BofA. Species migrating at positions expected for SpoIVFA, SpoIVFA dimer, and complex between SpoIVFA and full-length BofA are indicated. The complex, if present, is also identified with a dot (•) next to the sample treated only with Cu. A double star (**) indicates a cross-reacting protein. The position of migration of protein molecular weight (MW) markers is shown. Representative results from at least two biological replicates are shown. (**B**) Model highlighting residues capable of disulfide crosslinking when substituted with Cys. At left is a view showing only the BofA C-terminal region (starting with residue 55) and the SpoIVFA TMS (residues 71-92) oriented as in Figure 2B. Residues in proximity based on crosslinking are labeled, and the distance (Å) between their C_β_ atoms predicted by the previous model (8) (Table S1) is indicated. At right is a rotated view to show the predicted distance between C_β_ atoms of SpoIVFA V84 and BofA T65.

Full-length BofA coexpressed with SpoIVFA inhibits SpoIVFB cleavage of Pro-σ^K^(1–127) slightly better than GFPΔ27BofA (8). GFPΔ27BofA lacks the N-terminal 27 residues that include TMS1 of full-length BofA. To test whether full-length BofA forms disulfide crosslinks with SpoIVFA similar to MBPΔ27BofA, we coexpressed single-Cys variants of full-length BofA and SpoIVFA with Cys-less variants of SpoIVFB and Pro-σ^K^ in *E. coli* and performed disulfide crosslinking experiments. We observed a species migrating at ~39 kDa, the predicted molecular weight of SpoIVFA (29.6 kDa) crosslinked to full-length BofA (9.6 kDa), upon Cu treatment of *E. coli* coexpressing V84C SpoIVFA with V81C BofA (Fig. 4A, lane 5) or T65C BofA (lane 8), or coexpressing A88C SpoIVFA with I86C BofA (lane 14). These results provide evidence that the same two C-terminal regions of full-length BofA are proximal to the C-terminal part of the SpoIVFA TMS as we observed for MBPΔ27BofA (Fig. 3). Interestingly, treatment with Cu followed by DTT diminished the intensity of the ~39 kDa species observed for V84C SpoIVFA coexpressed with V81C BofA (Fig. 4A, lane 6), indicating partial reversal of the crosslink. This result differs from the corresponding result with V81C MBPΔ27BofA (Fig. 3A, lane 15). In contrast, DTT appeared to partially reverse the other two cross-links between MBPΔ27BofA and SpoIVFA (Fig. 3A, lanes 3 and 24), but not between full-length BofA and SpoIVFA (Fig. 4A, lanes 9 and 15). These results demonstrate differences between disulfide crosslinked protein complexes in terms of crosslink reversal by DTT after TCA precipitation, resuspension in SDS-containing buffer, and warming at 37°C for 7 min, which we do not understand.

Figure 4B highlights the residues that our results show can engage in disulfide crosslinking upon substitution with Cys and treatment of cells with oxidant. The predicted distances between C_β_ atoms involved in crosslinking, based on the previous model (8) (Fig. 2; Table S1), are shown for the highlighted residues. All the distances, except the 4.5 Å distance between SpoIVFA A88 and BofA I86, exceed the 5.4 Å length of a typical C_β_−S−S−C_β_ disulfide bond. We attribute the observed crosslinking over the range of predicted distances from 4.5 Å to 8.7 Å to model uncertainty, conformational fluctuations, and/or favorable side chain orientations. Conversely, unfavorable side chain orientations and/or model uncertainty may explain failure to observe crosslinks, despite predicted proximity. For most of the other combinations of single-Cys variants with substitutions in the MBPΔ27BofA C-terminal region and the SpoIVFA TMS, we observed little or no crosslinking (Fig. S2). A notable exception was the combination of S87C SpoIVFA and I82C MBPΔ27BofA (lane 13). This combination of residues is also highlighted in Figure 4B.

One combination yielded a very unexpected result; H65C SpoIVFA (with a single-Cys predicted to be near the cytoplasmic face of the inner membrane when expressed in *E. coli*) appeared to crosslink weakly with T65C MBPΔ27BofA (with a single-Cys predicted to be near the middle of the inner membrane) (lane 47). Although there are other possible explanations for the species migrating at ~75 kDa, we explored crosslinking conditions that were more stringent in order to eliminate such weak signals.

### Oxidant titration reveals the relative ability of disulfide crosslinks to form between the BofA C-terminal region and the SpoIVFA transmembrane segment

**Fig 5:**
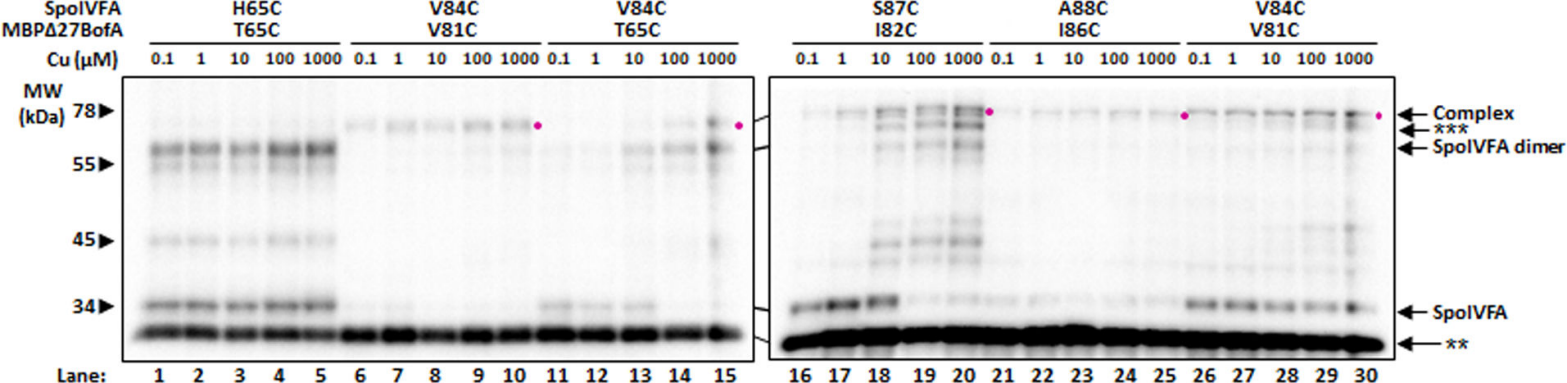
Disulfide crosslinking at different oxidant concentrations. Anti-SpoIVFA immunoblots of disulfide crosslinking between SpoIVFA and MBPΔ27BofA variants. The variants lack cysteine residues except at the indicated position. Variants were coexpressed in *E. coli,* and the cells were treated with oxidant Cu^2+^(phenanthroline)_3_ (Cu) at the indicated concentrations for 5 min to promote disulfide crosslinking. Species migrating at positions expected for SpoIVFA, SpoIVFA dimer, and complex between SpoIVFA and MBPΔ27BofA are indicated. The complex, if present, is also identified with a dot (•) next to the sample treated with 1000 µM Cu. A double star (**) indicates a cross-reacting protein. A triple star (***) denotes species migrating slightly faster than complex, which are most obvious in lanes 18–20 and may be due to partial degradation of SpoIVFA and/or MBPΔ27BofA variants. The position of migration of protein molecular weight (MW) markers is shown on the blot at left. Migration was slightly less on that gel; hence, the lines between the blots indicate their alignment. Representative results from at least two biological replicates are shown.

In the disulfide crosslinking experiments presented so far (Fig. 3 and 4A; Fig. S2), we treated cells with 1 mM Cu for 60 min. To explore conditions that might eliminate weak signals and allow comparison of strong signals, we treated cells with a 10-fold dilution series of Cu for 5 min (Fig. 5; Fig. S3A). We observed little or no crosslinking of H65C SpoIVFA to T65C MBPΔ27BofA (lanes 1-5), whereas V84C SpoIVFA crosslinked to V81C MBPΔ27BofA at all [Cu] from 1 mM to 0.1 µM (lanes 6–10, 26–30). Interestingly, V84C SpoIVFA crosslinked to T65C MBPΔ27BofA at 100 µM Cu, but we observed very little or no signal at ≤10 µM Cu (lanes 11–15), revealing less ability to form this crosslink than the one between V84C SpoIVFA and V81C MBPΔ27BofA. The other two combinations we tested, S87C SpoIVFA to I82C MBPΔ27BofA (lanes 16-20) and A88C SpoIVFA to I86C MBPΔ27BofA (lanes 21–25), exhibited similar crosslinking ability as V84C SpoIVFA and V81C MBPΔ27BofA. Hence, all three crosslinks between a Cys residue near the C-terminal end of BofA and one near the C-terminal end of the predicted SpoIVFA TMS formed more readily than the crosslink between T65C MBPΔ27BofA and V84C SpoIVFA, which, in turn, formed more readily than the unexpected ~75 kDa species observed for T65C MBPΔ27BofA and H65C SpoIVFA.

**Fig 6:**
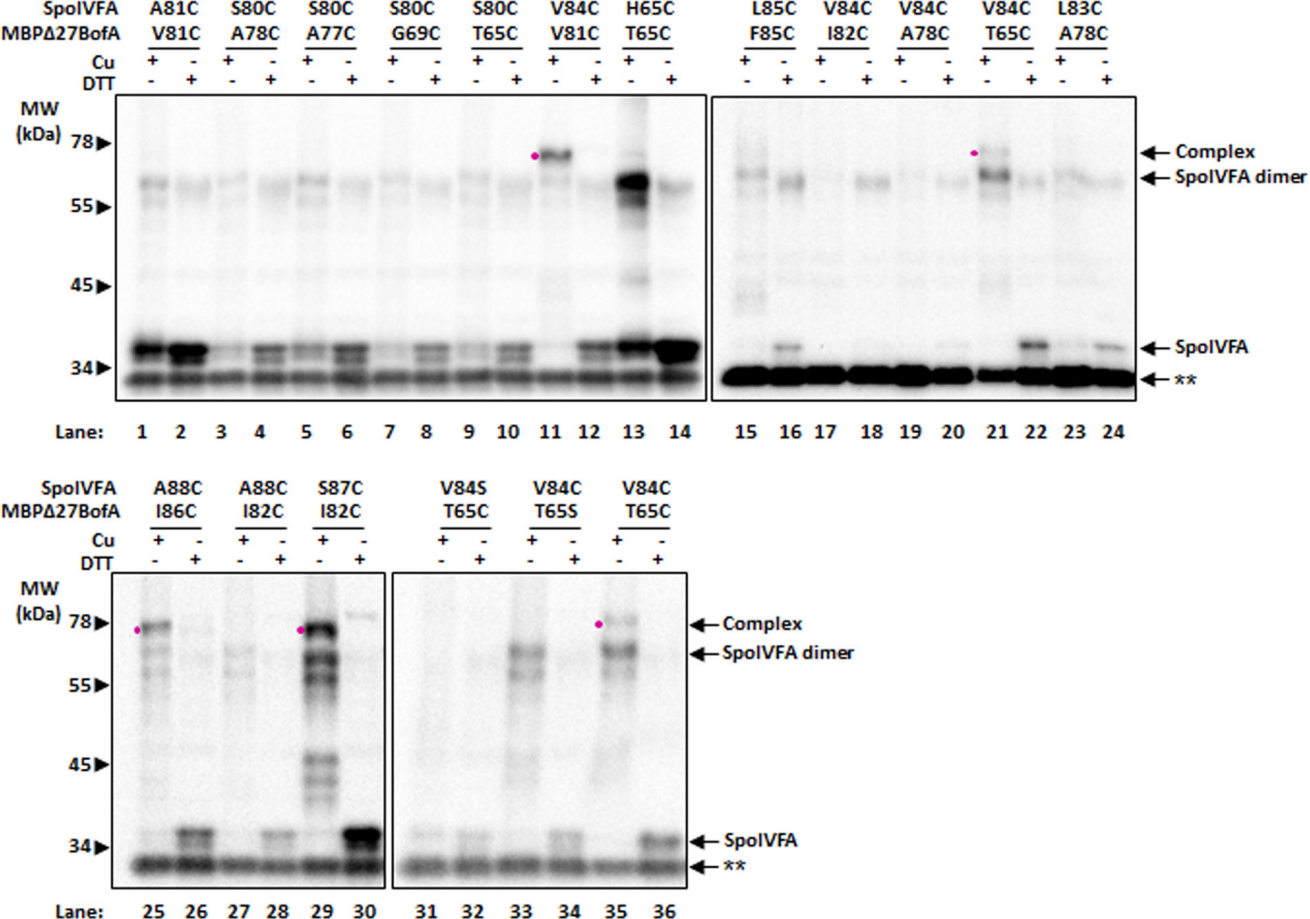
Disulfide crosslinking under stringent conditions. Anti-SpoIVFA immunoblots of crosslinking between SpoIVFA and MBPΔ27BofA variants. Crosslinking was performed as described in the Fig. 5 legend, except cells were treated with oxidant Cu^2+^(phenanthroline)_3_ (Cu) (100 µM) for 5 min to promote disulfide crosslinking. Species migrating at positions expected for SpoIVFA, SpoIVFA dimer, and complex between SpoIVFA and MBPΔ27BofA are indicated. The complex, if present, is also identified with a dot (•) next to the sample treated with 100 µM Cu. A double star (**) indicates a cross-reacting protein. The position of migration of protein molecular weight (MW) markers is shown. Representative results from at least two biological replicates are shown.

To determine whether any other combinations of single-Cys variants formed a disulfide crosslink as readily as T65C MBPΔ27BofA and V84C SpoIVFA, we treated cells coexpressing the protein variants with 100 µM Cu for 5 min (Fig. 6; Fig. S3B). Under these stringent conditions, we observed little or no crosslinking of T65C MBPΔ27BofA to H65C SpoIVFA (lane 13) or T65S MBPΔ27BofA to V84C SpoIVFA (lane 33), or any combinations other than those shown in Fig. 5. Hence, despite weak signals suggestive of crosslinking for some of the other combinations of single-Cys variants under less stringent conditions (Fig. S2), the weak signals did not persist under stringent conditions (Fig. 6; Fig. S3B). Therefore, we cannot be confident that the weak signals suggestive of crosslinking in Fig. S2 reflect proximity.

### Comparison with alternate models of SpoIVFB in complex with BofA, SpoIVFA, and Pro-σ^K^

**Fig 7:**
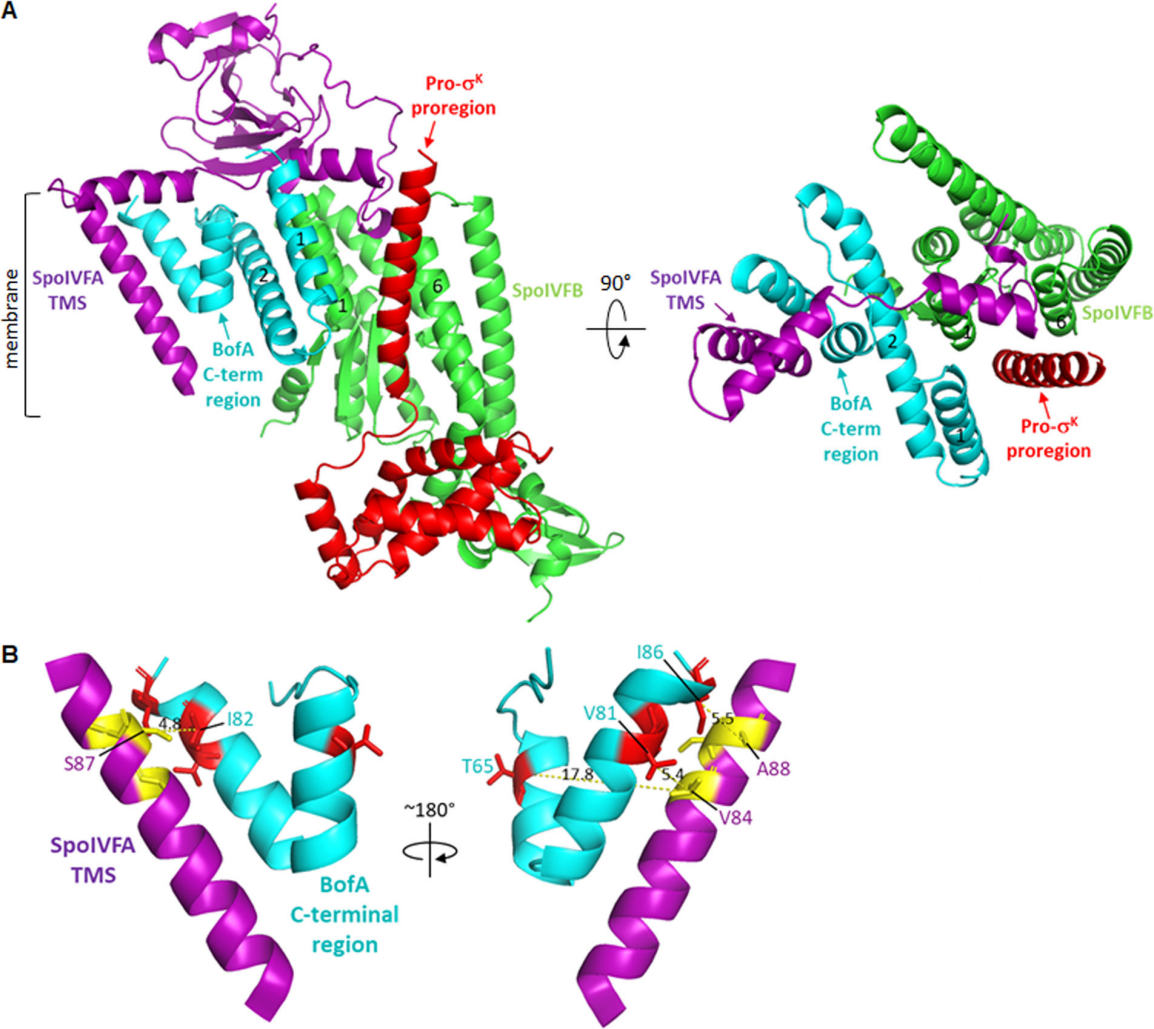
AlphaFold-2 Multimer version 2 structural prediction of SpoIVFB in complex with BofA and parts of SpoIVFA and Pro-σ^K^. (**A**) Model overview. At left is a side view of SpoIVFB with a relatively narrow opening between transmembrane segment 1 (TMS1) and TMS6, and part of SpoIVFA looped into the opening. BofA and the Pro-σ^K^ Proregion are excluded from the SpoIVFB active site cleft. At right is a top view showing the SpoIVFB membrane domain with BofA, SpoIVFA residues 70-135, and Pro-σ^K^ residues 1–25. The SpoIVFA TMS is predicted to be near only part of the BofA C-terminal region. (**B**) Model highlighting residues capable of disulfide crosslinking when substituted with Cys. At left is a view showing only the BofA C-terminal region (starting with residue 55) and the SpoIVFA TMS (residues 71-92) oriented as in panel A at left. Residues in proximity based on crosslinking are labeled, and the distance (Å) between their C_β_ atoms predicted by the AlphaFold-2 Multimer version 2 model (Table S1) is indicated. At right is a rotated view to show the predicted distance between C_β_ atoms of the other residues in proximity based on crosslinking.

We initially used AlphaFold-2 Multimer version 2 to predict the structure of the four-protein complex. We included only the N-terminal half of Pro-σ^K^ (residues 1–127), since its C-terminal half is dispensable for cleavage by SpoIVFB (51) (Fig. 7A; Fig. S4). As expected, confidence in the local structure (pLDDT scores) varied, as indicated by colors in the bottom part of Fig. S4. Notably, the unusual structure of the BofA C-terminal region is predicted with very high confidence. The overall confidence in the predicted structure of the complex (pTM = 0.73) is >0.5, suggesting that it might be correct, and the accuracy of the predicted subunit interfaces (ipTM = 0.67) is between 0.6 and 0.8, a gray zone in terms of confidence. In the model, the N-terminal 69 residues of SpoIVFA were disordered and did not interact with the other proteins; hence, those residues are not shown in Fig. 7A and Fig. S4. Strikingly, the model does not predict that BofA TMS2 is in the SpoIVFB active site cleft, as suggested by previous disulfide crosslinking results (8). Rather, the SpoIVFB active site cleft is predicted to be narrower, and neither BofA TMS2 nor the Proregion of Pro-σ^K^ occupies the cleft (Fig. 7A). Importantly, SpoIVFA V84 is not in proximity to BofA T65 (17.8 Å between C_β_ atoms) (Fig. 7B; Table S1), which is inconsistent with our disulfide crosslinking results (Fig. 3 to 6). In contrast, SpoIVFA V84 is closer to BofA T65 (8.0 Å between C_β_ atoms) in the previous model (Fig. 4B; Table S1). In both models, SpoIVFA C_β_ atoms of residues V84, S87, and A88 are within 4.5-8.7 Å of BofA C_β_ atoms of residues V81, I82, and I86, respectively (Table S1).

To investigate whether AlphaFold would generate structural predictions with BofA TMS2 in the SpoIVFB active site cleft, as suggested by our previous studies (8) (Fig. 2A), we predicted the *B. subtilis* four-protein complex five more times using AlphaFold-2 Multimer version 2 with random seeds and five times each with AlphaFold-2 Multimer version 3 and AlphaFold-3. None of the predictions placed BofA TMS2 in the SpoIVFB active site cleft. Interestingly, AlphaFold-3 was more confident in modeling the SpoIVFA N-terminal region and predicted a different configuration of BofA TMS1 relative to the Proregion of Pro-σ^K^ (Fig. S5). We also predicted the four-protein complex for three spore-forming bacteria distantly related to *B. subtilis* phylogenomically. None of the predictions placed BofA TMS2 in the SpoIVFB active site cleft. The orthologous protein sequences of *Alkalihalobacillus halodurans*, a distant relative in the same family (Bacillaceae), generated a very similar top-ranked model and Predicted Aligned Error (PAE) plot as *B. subtilis* (Fig. S6). The more distant relatives *Paenibacillus polymyxa* (family Paenibacillaceae) and *Kyrpidia tusciae* (family Alicyclobacillaceae) generated predictions with less confidence (Fig. S6B; Table S2). Interestingly, the top-ranked *P. polymyxa* model predicted a different configuration of BofA and SpoIVFA relative to SpoIVFB, which allowed the Pro-σ^K^ Proregion to form an extended β-sheet with the SpoIVFB membrane reentrant loop in its active site cleft (Fig. S6A), poised for cleavage.

## DISCUSSION

Our results provide evidence that two parts of the BofA C-terminal region are in proximity to the SpoIVFA TMS. These findings support the prediction that the BofA C-terminal region adopts an unusual structure within the membrane, which interacts with the SpoIVFA TMS to block SpoIVFB cleavage of Pro-σ^K^ (8). A disulfide crosslink between T65C MBPΔ27BofA and V84C SpoIVFA provides support for our previous model that the BofA TMS2 could bind the active site cleft of SpoIVFB. However, this crosslink formed less readily at low oxidant concentration than several other crosslinks we observed, and the presence of BofA’s TMS2 in the active site cleft was not predicted by AlphaFold-2 or AlphaFold-3 using multiple homologs of the four-protein complex. We propose that two parts of the BofA C-terminal region interact with the SpoIVFA TMS within the outer forespore membrane of sporulating *B. subtilis* and related species that encode SpoIVFA, blocking SpoIVFB activity until forespore signaling relieves inhibition. We discuss the implications of our findings and our proposal in relation to previous work and future directions.

### The BofA C-terminal region adopts an unusual structure within the membrane

The BofA C-terminal region was initially proposed to form a third TMS based on hydropathy analysis (31). Subsequent membrane topological analysis involving fusions of *bofA* to *lacZ* and *phoA* at several different positions expressed in *E. coli* suggested instead that the BofA C-terminal region was exposed in the periplasm (52). The authors did not rule out the possibility that part of the BofA C-terminal region loops into the membrane. Presumably, attachment of alkaline phosphatase (the *phoA* product) to such a loop could cause mislocalization to the periplasm. Recent computational modeling used predicted intra-chain contacts based on co-evolutionary couplings to constrain a structural model of BofA (8). The model predicted that BofA contains two TMSs and a membrane-embedded C-terminal region consisting of two short α-helices connected by a turn. The turn is formed by a G(L, I, or V)PG motif that is highly conserved among BofA orthologs. The N- and C-termini of this unusual structure would be at or near the membrane surface, with the region in between looping into the membrane, similar to the possibility mentioned by Varcamonti et al. (52). Importantly, the membrane-embedded BofA C-terminal region was predicted to interact with SpoIVFA, which has a single TMS; hence, the computational model focused our effort to identify proximity between the two proteins using a disulfide crosslinking approach (48).

### Interaction between the BofA C-terminal tip and SpoIVFA within the membrane appears to be important for the inhibition of SpoIVFB

Mutational analysis suggested that the BofA C-terminal region is important for its function or stability during *B. subtilis* sporulation. BofA is 87 residues long and mutations causing G75E or G75R substitutions interfered with the inhibition of SpoIVFB (31), bypassing its normal dependence on forespore signaling (29). Notably, G75 is in the highly conserved motif 72-GIPG-75 predicted to form a turn in the *B. subtilis* BofA C-terminal region (8). Replacement of the four C-terminal residues of BofA (QFII) with RILSAGR also interfered with SpoIVFB inhibition (31), as did deletion of three or four residues from the C-terminal end of BofA (52). These studies could not distinguish between interference with BofA function or stability since antibodies able to detect BofA and its variants were not (and still are not) available.

A recent study showed that the C-terminal tip of BofA is important for its function in SpoIVFB inhibition (8). *E. coli* was engineered to synthesize functional variants of BofA, SpoIVFA, SpoIVFB, and Pro-σ^K^ that were detectable by immunoblot. GFPΔ27BofA lacking TMS1 is functional in sporulating *B. subtilis* (49) and in the *E. coli* system (8), and is detectable by anti-GFP antibodies. GFPΔ27BofA variants with single-Ala substitutions (F85A, I86A, or I87A) had little effect on the inhibition of SpoIVFB cleavage of Pro-σ^K^. However, deleting those three residues or substituting Ala for all three nearly eliminated inhibition, despite normal accumulation of the variants. Although the majority of 69 BofA orthologs examined have three hydrophobic residues at their C-terminal tip, one-third have one or more charged, polar, or Gly residue, indicating a hydrophobic patch is not a highly conserved feature. Nevertheless, we conclude that hydrophobic residues at the C-terminal tip of *B. subtilis* BofA are important for its function in SpoIVFB inhibition in the *E. coli* system (8) and likely in sporulating *B. subtilis* (31, 52).

Our disulfide crosslinking results provide evidence that the BofA C-terminal tip is in proximity to the SpoIVFA TMS. Since a disulfide crosslink between protein residue C_β_ atoms (i.e., C_β_−S−S−C_β_) is typically 5.4 Å, our results imply that residues V81, I82, and I86 at the *B. subtilis* BofA C-terminal tip are close enough to residues V84, S87, and A88 in the C-terminal part of the SpoIVFA TMS, respectively, for side chain interactions to occur. Both the structural model proposed previously (8) and the models we generated using AlphaFold predict that a short α-helix at the BofA C-terminal tip lies proximal to the SpoIVFA TMS (Fig. 4B and 7B; Fig. S5). These two α-helices have hydrophobic faces that likely interact *via* van der Waals forces (Fig. S7). The interaction may help SpoIVFA recruit BofA to the SpoIVFB inhibition complex, based on evidence that SpoIVFA plays the key role in forming the complex and localizing it to the outer forespore membrane of sporulating *B. subtilis* (49). It was also shown that SpoIVFA coimmunoprecipitated with GFP-BofA in the absence of SpoIVFB, consistent with a direct interaction between SpoIVFA and BofA. Disruption of this interaction presumably contributes to the loss of SpoIVFB inhibition observed for BofA G75 substitutions (29, 31), and an addition to (31), deletions from (8, 52), and substitutions within (8) the BofA C-terminal tip.

### Interaction between a second part of the BofA C-terminal region and the SpoIVFA transmembrane segment appears to be important for the inhibition of SpoIVFB

In addition to disulfide crosslinks between the BofA C-terminal tip and the SpoIVFA TMS, we observed a crosslink between T65C MBPΔ27BofA (or full-length T65C BofA) and V84C SpoIVFA (Fig. 3A and 4). This crosslink had less ability to form at low oxidant concentration than the three crosslinks we observed between the BofA C-terminal tip and the SpoIVFA TMS (Fig. 5 and 6). The low crosslinking ability may be due to less favorable orientations of T65C MBPΔ27BofA and V84C SpoIVFA, and/or less favorable conformational fluctuations of the regions containing those residues. The distance of 8.0 Å between BofA T65 and SpoIVFA V84 C_β_ atoms predicted by the previous model suggested that it might be possible to observe a disulfide crosslink (Fig. 4B), but the C_β_ distance of 17.8 Å predicted by the AlphaFold-2 Multimer version 2 model suggested that the crosslink would not be observed (Fig. 7B). Hence, the disulfide crosslinking result is consistent with our previous model, suggesting the AlphaFold models are incorrect. However, it is also possible that this weaker crosslink is capturing a transient conformation of the complex or is possibly not physiologically relevant.

We generated many additional models using different versions of AlphaFold to predict structures of the *B. subtilis* SpoIVFB inhibition complex, as well as that of other spore-forming bacteria. None of the models agreed with the previous model (8) by placing BofA TMS2 in the SpoIVFB active site cleft. Neither did any of the models reduce the distance between BofA T65 and SpoIVFA V84 C_β_ atoms sufficiently to be consistent with the disulfide crosslinking we observed (Fig. 3A and 4). Most of the models were similar to that for *B. subtilis*, but those for *P. polymyxa* predicted a different configuration of BofA and SpoIVFA relative to SpoIVFB (Fig. S6A). The predicted complex would not likely inhibit SpoIVFB activity, since the Proregion of Pro-σ^K^ is positioned at the enzyme active site as if engaged for cleavage (22). In contrast, the models for *B. subtilis* and the other two sporeformers predict that a segment of SpoIVFA between its TMS and C-terminal domain blocks the gate between SpoIVFB TMS1 and TMS6 proposed by Ramirez-Guadiana et al. (32), preventing access of the Pro-σ^K^ Proregion to the enzyme active site (Fig. S6). An attractive feature of these models is the apparent ease with which SpoIVFB inhibition could be relieved, since SpoIVB and CtpB cleave SpoIVFA in the segment that obstructs access to the proposed SpoIVFB active site gate (32–34, 36, 37). However, many predictions that can be inferred from the AlphaFold models remain to be experimentally tested.

Our previous *in vivo* studies indicate that alanine substitutions at T64A, N61A, and N48 in BofA interfere with SpoIVFB inhibition (8). All three residues are highly conserved in BofA orthologs. We therefore proposed that *B. subtilis* BofA N48, N61, and T64 function as a critical structural domain that includes TMS2 and the membrane-embedded C-terminal region. Our previous model predicted that this domain interacts with SpoIVFA and SpoIVFB. The results presented here demonstrate proximity between the SpoIVFA TMS and a part of the BofA C-terminal region, providing support for the critical role of this structural domain.

### Inhibition of SpoIVFB in species related to *B. subtilis*

The phylogenomic distribution of SpoIVFB is broader than that of BofA, which in turn is broader than that of SpoIVFA, raising interesting questions about SpoIVFB inhibition in relatives of *B. subtilis*. In a recent study of 180 diverse firmicute genomes, *spoIVFB* orthologs were surprisingly widespread, with 56 in nonsporeformers and 75 among the 76 sporeformers examined (53). The role of SpoIVFB in nonsporeformers is unknown. The sporeformers included several *Bacilli* and *Clostridia* that are human or plant pathogens. Orthologs of *bofA* were found in 62 spore-forming *Bacilli* and *Clostridia*, and in 21 nonsporeformers. In contrast, *spoIVFA* orthologs were recognized in 25 spore-forming *Bacilli*, but in only two spore-forming *Clostridia* and six nonsporeformers. These observations raise questions about whether SpoIVFB activity is regulated in bacteria apparently lacking SpoIVFA and/or BofA, and if so, how.

Our results provide new targets for developing modulators of SpoIVFB inhibition. Modulators could inhibit or enhance the interactions between the BofA C-terminal region and the SpoIVFA TMS we have uncovered, thus weakening or strengthening, respectively, the inhibition of SpoIVFB activity. The most apparent application would be controlling the sporulation of *Bacilli* such as the human pathogens *Bacillus anthracis* and *Bacillus cereus*, and the plant pathogen *Bacillus thuringiensis*. The endospores formed by these species and other spore-forming *Bacilli* and *Clostridia* are dormant and resist environmental insults, enhancing survival and making them difficult to eradicate (53–56). Efforts toward controlling sporulation of the human pathogens *Clostridium botulinum*, *Clostridium perfringens*, and *Clostridium tetani* could focus on determining whether BofA alone inhibits SpoIVFB, since these *Clostridia* lack *spoIVFA* orthologs (53). For example, clostridial SpoIVFB and Pro-σ^K^ with or without BofA could be coexpressed in *E. coli*, and Pro-σ^K^ cleavage could be measured. If BofA inhibits SpoIVFB activity, it would motivate purification of the protein complex for structural analysis to guide rational design of inhibitors.

## MATERIALS AND METHODS

### Plasmids and primers

Plasmids and primers used in this study are listed in Tables S3 and S4, respectively. Plasmids were cloned in *E. coli* strain DH5α (57). Plasmid DNA sequences were verified by Sanger (Michigan State University Research Technology Support Facility) and whole-plasmid (Plasmidsaurus) sequencing.

### Induction of protein production in *E. coli*

Plasmids were electroporated into *E. coli* strain BL21(DE3) (Novagen) and transformants were selected on Luria-Bertani (LB) agar supplemented with kanamycin sulfate (50 µg/mL). Four or five colonies were inoculated into LB medium containing 50 µg/mL kanamycin sulfate and incubated at 37°C with shaking at 200 rpm. An inoculum (200 µL) from an overnight culture was transferred into 10 mL of LB medium with 50 µg/mL kanamycin sulfate. These cultures were incubated at 37°C with shaking at 250 rpm until they reached an optical density of 60–80 Klett units. Isopropyl β-D-thiogalactopyranoside (IPTG) (0.5 mM) was added to induce protein production for 2 h. Equivalent amounts of cells based on optical density measurements were centrifuged at 12,000 × *g* for 1 min, and the cell pellets were used in experiments.

### Disulfide crosslinking

Disulfide crosslinking was performed as described previously (58). Briefly, after induction of protein production, cell pellets were mixed with chloramphenicol (200 µg/mL) and 2-phenanthroline (3 mM), the samples were centrifuged at 12,000 × *g* for 1 min, the supernatants were discarded, and the cells were suspended in 10 mM Tris-HCl (pH 8.1) and 3 mM 2-phenanthroline. The samples were again centrifuged, the supernatants were discarded, and the cells were suspended in 10 mM Tris-HCl (pH 8.1). Sample aliquots were treated with Cu^2+^(phenanthroline)_3_ (0.1–1,000 µM) to promote disulfide crosslinking or with 3 mM 2-phenanthroline as a control for 5 or 60 min at 37°C and subsequently treated with neocuproine (12.5 mM) for 5 min at 37°C. The cells were lysed, and proteins were precipitated by adding trichloroacetic acid (5%) with mixing by inversion every 5 min for 30 min on ice. After centrifugation at 12,000 × *g* for 15 min at 4°C to sediment the proteins, the supernatants were discarded, and the pellets were washed with ice-cold acetone. After centrifugation at 12,000 × *g* for 5 min at 4°C, the supernatants were discarded, and the pellets were air-dried for 5 min at room temperature. The pellets were dissolved in buffer solution (100 mM Tris-HCl [pH 7.5], 1.5% SDS, 5 mM EDTA, 25 mM *N*-ethylmaleimide) by slowly pipetting up and down every 10 min for 30 min at room temperature. Sample aliquots were mixed with an equal volume of sample buffer (25 mM Tris-HCl [pH 6.8], 2% SDS, 10% glycerol, 0.015% bromophenol blue) with or without 100 mM DTT and warmed at 37°C for 10 min prior to immunoblot analysis.

### Inhibition of Pro-σ^K^(1-127) cleavage

After induction of protein production, the extracts of cell pellets were prepared as described previously (59), then subjected to immunoblot analysis to determine the amounts of Pro-σ^K^(1-127) and cleavage product.

### Immunoblot analysis

Samples were subjected to immunoblot analysis as described previously (60), except with some modifications. Briefly, proteins were separated by SDS-PAGE using discontinuous (5% stacking, 10% resolving) polyacrylamide gels and Tris-Tricine electrode buffer (0.1 M Tris, 0.1 M Tricine, 0.1% SDS, pH 8.3). Separation was monitored using SeeBlue Plus2 Prestained Standard (Invitrogen). Proteins were electroblotted to Immobilon-P membranes (Millipore), and blots were incubated in a solution of 5% nonfat dry milk in TBST (20 mM Tris-HCl at pH 7.5, 0.5 M NaCl, 0.1% Tween 20) for 1 h at 25°C with shaking to block nonspecific binding of antibodies. Blots were then incubated with rabbit polyclonal antiserum against SpoIVFA (60) (1:3,000) or MBP (catalog number E8030S; NEB) (1:10,000), or mouse monoclonal anti-penta-His-HRP conjugate (catalog number 34460; Qiagen) (1:10,000) diluted in TBST containing 2% milk overnight at 4°C with shaking. To detect the anti-SpoIVFA and anti-MBP antibodies, a goat anti-rabbit HRP-linked antibody (catalog number 170-6515; Bio-Rad) (1:10,000) diluted in TBST with 2% milk was incubated with blots for 1 h at 25°C with shaking. Signals were generated using the Western Lightning Plus ECL reagents (PerkinElmer) and detected using a ChemiDoc MP imaging system (Bio-Rad). To determine the Pro-σ^K^(1–127) cleavage ratio, unsaturated signals were quantified using the Image Lab 5.1 software (Bio-Rad) lane and bands tool.

### AlphaFold structural predictions

AlphaFold predictions were run using AlphaFold-3 *via* the online web portal at https://alphafoldserver.com/welcome (61), or by using AlphaFold-2 Multimer (62) (version 2 or version 3 as indicated in figures/tables) as implemented in localcolabfold (63). All predictions utilized the PDB templates option and were otherwise run using default parameters. All AlphaFold-3 predictions were run, including a single Zn^2+^ ion to occupy the SpoIVFB active site.

## SUPPLEMENTAL MATERIAL

Supplemental tables and figures jb.00186-25-s0001.pdf Tables S1 to S4; Figures S1 to S7.

ASM does not own the copyrights to Supplemental Material that may be linked to, or accessed through, an article. The authors have granted ASM a non-exclusive, world-wide license to publish the Supplemental Material files. Please contact the corresponding author directly for reuse.

## References

[1] Brown MS, Ye J, Rawson RB, Goldstein JL. 2000. Regulated intramembrane proteolysis: a control mechanism conserved from bacteria to humans. Cell 100:391–398. doi:10.1016/s0092-8674(00)80675-3 10693756

[2] Urban S. 2013. Mechanisms and cellular functions of intramembrane proteases. Biochim Biophys Acta 1828:2797–2800. doi:10.1016/j.bbamem.2013.07.001 23831604

[3] Lichtenthaler SF, Lemberg MK, Fluhrer R. 2018. Proteolytic ectodomain shedding of membrane proteins in mammals-hardware, concepts, and recent developments. EMBO J 37:e99456. doi:10.15252/embj.201899456 29976761 PMC6068445

[4] Urban S. 2009. Making the cut: central roles of intramembrane proteolysis in pathogenic microorganisms. Nat Rev Microbiol 7:411–423. doi:10.1038/nrmicro2130 19421188 PMC2818034

[5] Hizukuri Y, Oda T, Tabata S, Tamura-Kawakami K, Oi R, Sato M, Takagi J, Akiyama Y, Nogi T. 2014. A structure-based model of substrate discrimination by a noncanonical PDZ tandem in the intramembrane-cleaving protease RseP. Structure 22:326–336. doi:10.1016/j.str.2013.12.003 24389025

[6] Bolduc DM, Montagna DR, Gu YL, Selkoe DJ, Wolfe MS. 2016. Nicastrin functions to sterically hinder γ-secretase-substrate interactions driven by substrate transmembrane domain. Proc Natl Acad Sci USA 113:E509–E518. doi:10.1073/pnas.1512952113 26699478 PMC4747693

[7] Miyake T, Hizukuri Y, Akiyama Y. 2020. Involvement of a membrane-bound amphiphilic helix in substrate discrimination and binding by an Escherichia coli S2P peptidase RseP. Front Microbiol 11:607381. doi:10.3389/fmicb.2020.607381 33329500 PMC7728848

[8] Olenic S, Heo L, Feig M, Kroos L. 2022. Inhibitory proteins block substrate access by occupying the active site cleft of Bacillus subtilis intramembrane protease SpoIVFB. eLife 11:e74275. doi:10.7554/eLife.74275 35471152 PMC9042235

[9] Beard HA, Barniol-Xicota M, Yang J, Verhelst SHL. 2019. Discovery of cellular roles of intramembrane proteases. ACS Chem Biol 14:2372–2388. doi:10.1021/acschembio.9b00404 31287658

[10] Kühnle N, Dederer V, Lemberg MK. 2019. Intramembrane proteolysis at a glance: from signalling to protein degradation. J Cell Sci 132:jcs217745. doi:10.1242/jcs.217745 31416853

[11] Rawson RB. 2013. The site-2 protease. Biochim Biophys Acta 1828:2801–2807. doi:10.1016/j.bbamem.2013.03.031 23571157

[12] Ye J. 2013. Roles of regulated intramembrane proteolysis in virus infection and antiviral immunity. Biochim Biophys Acta 1828:2926–2932. doi:10.1016/j.bbamem.2013.05.005 24099010 PMC3837687

[13] Kroos L, Akiyama Y. 2013. Biochemical and structural insights into intramembrane metalloprotease mechanisms. Biochim Biophys Acta 1828:2873–2885. doi:10.1016/j.bbamem.2013.03.032 24099006 PMC3793210

[14] Schneider JS, Glickman MS. 2013. Function of site-2 proteases in bacteria and bacterial pathogens. Biochim Biophys Acta 1828:2808–2814. doi:10.1016/j.bbamem.2013.04.019 24099002 PMC4097180

[15] Sineva E, Savkina M, Ades SE. 2017. Themes and variations in gene regulation by extracytoplasmic function (ECF) sigma factors. Curr Opin Microbiol 36:128–137. doi:10.1016/j.mib.2017.05.004 28575802 PMC5534382

[16] Kristensen SS, Diep DB, Kjos M, Mathiesen G. 2023. The role of site-2-proteases in bacteria: a review on physiology, virulence, and therapeutic potential. Microlife 4:uqad025. doi:10.1093/femsml/uqad025 37223736 PMC10202637

[17] Sun L, Li X, Shi Y. 2016. Structural biology of intramembrane proteases: mechanistic insights from rhomboid and S2P to γ-secretase. Curr Opin Struct Biol 37:97–107. doi:10.1016/j.sbi.2015.12.008 26811996

[18] Cho S, Baker RP, Ji M, Urban S. 2019. Ten catalytic snapshots of rhomboid intramembrane proteolysis from gate opening to peptide release. Nat Struct Mol Biol 26:910–918. doi:10.1038/s41594-019-0296-9 31570873 PMC6858540

[19] Yang G, Zhou R, Zhou Q, Guo X, Yan C, Ke M, Lei J, Shi Y. 2019. Structural basis of Notch recognition by human γ-secretase. Nature 565:192–197. doi:10.1038/s41586-018-0813-8 30598546

[20] Zhou R, Yang G, Guo X, Zhou Q, Lei J, Shi Y. 2019. Recognition of the amyloid precursor protein by human γ-secretase. Science 363:eaaw0930. doi:10.1126/science.aaw0930 30630874

[21] Imaizumi Y, Takanuki K, Miyake T, Takemoto M, Hirata K, Hirose M, Oi R, Kobayashi T, Miyoshi K, Aruga R, Yokoyama T, Katagiri S, Matsuura H, Iwasaki K, Kato T, Kaneko MK, Kato Y, Tajiri M, Akashi S, Nureki O, Hizukuri Y, Akiyama Y, Nogi T. 2022. Mechanistic insights into intramembrane proteolysis by E. coli site-2 protease homolog RseP. Sci Adv 8:eabp9011. doi:10.1126/sciadv.abp9011 36001659 PMC9401612

[22] Orlando MA, Pouillon HJT, Mandal S, Kroos L, Orlando BJ. 2024. Substrate engagement by the intramembrane metalloprotease SpoIVFB. Nat Commun 15:8276. doi:10.1038/s41467-024-52634-6 39419996 PMC11486902

[23] Asahi K, Hirose M, Aruga R, Shimizu Y, Tajiri M, Tanaka T, Adachi Y, Tanaka Y, Kaneko MK, Kato Y, Akashi S, Akiyama Y, Hizukuri Y, Kato T, Nogi T. 2025. Cryo-EM structure of the bacterial intramembrane metalloprotease RseP in the substrate-bound state. Sci Adv 11:eadu0925. doi:10.1126/sciadv.adu0925 40009668 PMC11864173

[24] Devkota S, Zhou R, Nagarajan V, Maesako M, Do H, Noorani A, Overmeyer C, Bhattarai S, Douglas JT, Saraf A, Miao Y, Ackley BD, Shi Y, Wolfe MS. 2024. Familial Alzheimer mutations stabilize synaptotoxic γ-secretase-substrate complexes. Cell Rep 43:113761. doi:10.1016/j.celrep.2024.113761 38349793 PMC10941010

[25] Bhattarai S, Liu L, Wolfe MS. 2021. Discovery of aryl aminothiazole γ-secretase modulators with novel effects on amyloid β-peptide production. Bioorg Med Chem Lett 54:128446. doi:10.1016/j.bmcl.2021.128446 34767913 PMC9759287

[26] Cho S, Dickey SW, Urban S. 2016. Crystal structures and inhibition kinetics reveal a two-stage catalytic mechanism with drug design implications for rhomboid proteolysis. Mol Cell 61:329–340. doi:10.1016/j.molcel.2015.12.022 26805573 PMC4744120

[27] Yang G, Zhou R, Guo X, Yan C, Lei J, Shi Y. 2021. Structural basis of γ-secretase inhibition and modulation by small molecule drugs. Cell 184:521–533. doi:10.1016/j.cell.2020.11.049 33373587

[28] Sun G, Yang M, Jiang L, Huang M. 2021. Regulation of Pro-σ^K^ activation: a key checkpoint in Bacillus subtilis sporulation. Environ Microbiol 23:2366–2373. doi:10.1111/1462-2920.15415 33538382

[29] Cutting S, Oke V, Driks A, Losick R, Lu S, Kroos L. 1990. A forespore checkpoint for mother cell gene expression during development in B. subtilis. Cell 62:239–250. doi:10.1016/0092-8674(90)90362-i 2115401

[30] Cutting S, Roels S, Losick R. 1991. Sporulation operon spoIVF and the characterization of mutations that uncouple mother-cell from forespore gene expression in Bacillus subtilis. J Mol Biol 221:1237–1256. doi:10.1016/0022-2836(91)90931-u 1942049

[31] Ricca E, Cutting S, Losick R. 1992. Characterization of bofA, a gene involved in intercompartmental regulation of pro-σ^K^ processing during sporulation in Bacillus subtilis. J Bacteriol 174:3177–3184. doi:10.1128/jb.174.10.3177-3184.1992 1577688 PMC205984

[32] Ramírez-Guadiana FH, Rodrigues CDA, Marquis KA, Campo N, Barajas-Ornelas RDC, Brock K, Marks DS, Kruse AC, Rudner DZ. 2018. Evidence that regulation of intramembrane proteolysis is mediated by substrate gating during sporulation in Bacillus subtilis. PLoS Genet 14:e1007753. doi:10.1371/journal.pgen.1007753 30403663 PMC6242693

[33] Campo N, Rudner DZ. 2006. A branched pathway governing the activation of a developmental transcription factor by regulated intramembrane proteolysis. Mol Cell 23:25–35. doi:10.1016/j.molcel.2006.05.019 16818230

[34] Campo N, Rudner DZ. 2007. SpoIVB and CtpB are both forespore signals in the activation of the sporulation transcription factor σ^K^ in Bacillus subtilis. J Bacteriol 189:6021–6027. doi:10.1128/JB.00399-07 17557826 PMC1952037

[35] Cutting S, Driks A, Schmidt R, Kunkel B, Losick R. 1991. Forespore-specific transcription of a gene in the signal transduction pathway that governs Pro-σ^K^ processing in Bacillus subtilis. Genes Dev 5:456–466. doi:10.1101/gad.5.3.456 1900494

[36] Dong TC, Cutting SM. 2003. SpoIVB-mediated cleavage of SpoIVFA could provide the intercellular signal to activate processing of Pro-σ^K^ in Bacillus subtilis. Mol Microbiol 49:1425–1434. doi:10.1046/j.1365-2958.2003.03651.x 12940997

[37] Mastny M, Heuck A, Kurzbauer R, Heiduk A, Boisguerin P, Volkmer R, Ehrmann M, Rodrigues CDA, Rudner DZ, Clausen T. 2013. CtpB assembles a gated protease tunnel regulating cell-cell signaling during spore formation in Bacillus subtilis. Cell 155:647–658. doi:10.1016/j.cell.2013.09.050 24243021 PMC3808539

[38] Pan Q, Losick R, Rudner DZ. 2003. A second PDZ-containing serine protease contributes to activation of the sporulation transcription factor σ^K^ in Bacillus subtilis. J Bacteriol 185:6051–6056. doi:10.1128/JB.185.20.6051-6056.2003 14526016 PMC225033

[39] Zhou R, Kroos L. 2005. Serine proteases from two cell types target different components of a complex that governs regulated intramembrane proteolysis of pro-σ^K^ during Bacillus subtilis development. Mol Microbiol 58:835–846. doi:10.1111/j.1365-2958.2005.04870.x 16238631 PMC2361100

[40] Rudner DZ, Fawcett P, Losick R. 1999. A family of membrane-embedded metalloproteases involved in regulated proteolysis of membrane-associated transcription factors. Proc Natl Acad Sci USA 96:14765–14770. doi:10.1073/pnas.96.26.14765 10611287 PMC24722

[41] Yu Y-T, Kroos L. 2000. Evidence that SpoIVFB is a novel type of membrane metalloprotease governing intercompartmental communication during Bacillus subtilis sporulation. J Bacteriol 182:3305–3309. doi:10.1128/JB.182.11.3305-3309.2000 10809718 PMC94525

[42] Zhou R, Cusumano C, Sui D, Garavito RM, Kroos L. 2009. Intramembrane proteolytic cleavage of a membrane-tethered transcription factor by a metalloprotease depends on ATP. Proc Natl Acad Sci USA 106:16174–16179. doi:10.1073/pnas.0901455106 19805276 PMC2752518

[43] Eichenberger P, Fujita M, Jensen ST, Conlon EM, Rudner DZ, Wang ST, Ferguson C, Haga K, Sato T, Liu JS, Losick R. 2004. The program of gene transcription for a single differentiating cell type during sporulation in Bacillus subtilis. PLoS Biol 2:e328. doi:10.1371/journal.pbio.0020328 15383836 PMC517825

[44] Kroos L, Kunkel B, Losick R. 1989. Switch protein alters specificity of RNA polymerase containing a compartment-specific sigma factor. Science 243:526–529. doi:10.1126/science.2492118 2492118

[45] Akiyama K, Mizuno S, Hizukuri Y, Mori H, Nogi T, Akiyama Y. 2015. Roles of the membrane-reentrant β-hairpin-like loop of RseP protease in selective substrate cleavage. eLife 4:e08928. doi:10.7554/eLife.08928 26447507 PMC4597795

[46] Zoll S, Stanchev S, Began J, Skerle J, Lepšík M, Peclinovská L, Majer P, Strisovsky K. 2014. Substrate binding and specificity of rhomboid intramembrane protease revealed by substrate-peptide complex structures. EMBO J 33:2408–2421. doi:10.15252/embj.201489367 25216680 PMC4253528

[47] Liu X, Zhao J, Zhang Y, Ubarretxena-Belandia I, Forth S, Lieberman RL, Wang C. 2020. Substrate-enzyme interactions in intramembrane proteolysis: γ-secretase as the prototype. Front Mol Neurosci 13:65. doi:10.3389/fnmol.2020.00065 32508589 PMC7248309

[48] Olenic S, Kroos L. 2023. An optimized disulfide cross-linking protocol to determine interactions of proteins produced in Escherichia coli. STAR Protoc 4:101962. doi:10.1016/j.xpro.2022.101962 36566383 PMC9803820

[49] Rudner DZ, Losick R. 2002. A sporulation membrane protein tethers the pro-σ^K^ processing enzyme to its inhibitor and dictates its subcellular localization. Genes Dev 16:1007–1018. doi:10.1101/gad.977702 11959848 PMC152351

[50] Zhang Y, Luethy PM, Zhou R, Kroos L. 2013. Residues in conserved loops of intramembrane metalloprotease SpoIVFB interact with residues near the cleavage site in pro-σ^K^. J Bacteriol 195:4936–4946. doi:10.1128/JB.00807-13 23995631 PMC3807480

[51] Prince H, Zhou R, Kroos L. 2005. Substrate requirements for regulated intramembrane proteolysis of Bacillus subtilis pro-σ^K^. J Bacteriol 187:961–971. doi:10.1128/JB.187.3.961-971.2005 15659674 PMC545722

[52] Varcamonti M, Marasco R, Maurilio DF, Sacco M. 1997. Membrane topology analysis of the Bacillus subtilis BofA protein involved in pro-σ^K^ processing. Microbiology (Reading, Engl) 143:1053–1058. doi:10.1099/00221287-143-4-1053 9141672

[53] Galperin MY, Yutin N, Wolf YI, Vera Alvarez R, Koonin EV. 2022. Conservation and evolution of the sporulation gene set in diverse members of the Firmicutes. J Bacteriol 204:e0007922. doi:10.1128/jb.00079-22 35638784 PMC9210971

[54] Setlow P. 2014. Spore resistance properties. Microbiol Spectr 2. doi:10.1128/microbiolspec.TBS-0003-2012 26104355

[55] Shen A, Edwards AN, Sarker MR, Paredes-Sabja D. 2019. Sporulation and germination in clostridial pathogens. Microbiol Spectr 7. doi:10.1128/microbiolspec.gpp3-0017-2018 PMC6927485 31858953

[56] Setlow P, Christie G. 2023. New thoughts on an old topic: secrets of bacterial spore resistance slowly being revealed. Microbiol Mol Biol Rev 87:e0008022. doi:10.1128/mmbr.00080-22 36927044 PMC10304885

[57] Hanahan D. 1983. Studies on transformation of Escherichia coli with plasmids. J Mol Biol 166:557–580. doi:10.1016/s0022-2836(83)80284-8 6345791

[58] Olenic S, Buchanan F, VanPortfliet J, Parrell D, Kroos L. 2022. Conserved proline residues of Bacillus subtilis intramembrane metalloprotease SpoIVFB are important for substrate interaction and cleavage. J Bacteriol 204:e0038621. doi:10.1128/JB.00386-21 35007155 PMC8923169

[59] Zhou R, Kroos L. 2004. BofA protein inhibits intramembrane proteolysis of pro-σ^K^ in an intercompartmental signaling pathway during Bacillus subtilis sporulation. Proc Natl Acad Sci USA 101:6385–6390. doi:10.1073/pnas.0307709101 15087499 PMC404054

[60] Kroos L, Yu Y-TN, Mills D, Ferguson-Miller S. 2002. Forespore signaling is necessary for pro-σ^K^ processing during Bacillus subtilis sporulation despite the loss of SpoIVFA upon translational arrest. J Bacteriol 184:5393–5401. doi:10.1128/JB.184.19.5393-5401.2002 12218026 PMC135367

[61] Abramson J, Adler J, Dunger J, Evans R, Green T, Pritzel A, Ronneberger O, Willmore L, Ballard AJ, Bambrick J, et al.. 2024. Accurate structure prediction of biomolecular interactions with AlphaFold 3. Nature 630:493–500. doi:10.1038/s41586-024-07487-w 38718835 PMC11168924

[62] Evans R, O’Neill M, Pritzel A, Antropova N, Senior A, Green T, Žídek A, Bates R, Blackwell S, Yim J, Ronneberger O, Bodenstein S, Zielinski M, Bridgland A, Potapenko A, Cowie A, Tunyasuvunakool K, Jain R, Clancy E, Kohli P, Jumper J, Hassabis D. 2022. Protein complex prediction with AlphaFold-Multimer. bioRxiv. doi:10.1101/2021.10.04.463034

[63] Mirdita M, Schütze K, Moriwaki Y, Heo L, Ovchinnikov S, Steinegger M. 2022. ColabFold: making protein folding accessible to all. Nat Methods 19:679–682. doi:10.1038/s41592-022-01488-1 35637307 PMC9184281

