# Supplementary material for: Inhibitory proteins of Bacillus subtilis interact within the membrane to block intramembrane protease activity
Source: J Bacteriol. 2025 Oct 8;207(11):e00186-25. doi: 10.1128/jb.00186-25 (PMC12632252; doi:10.1128/jb.00186-25)
Supplement: Supplemental tables and figures — Tables S1 to S4; Figures S1 to S7. [file jb.00186-25-s0001.pdf]

SUPPLEMENTAL MATERIAL

Inhibitory proteins of *Bacillus subtilis* interact within the membrane to block intramembrane protease activity

Saikat Mandal[†], Alanah Soriano[†], Caroline Erpelding[#], Jackson Ruffner[#], Eric Smith[#], Benjamin J. Orlando, Lee Kroos*

Department of Biochemistry and Molecular Biology, Michigan State University, East Lansing, Michigan, USA

Running title: SpoIVFA and BofA interact to block SpoIVFB activity

[†]Equal contributions

[#]Equal contributions

*Correspondence: kroos@msu.edu

Table S1. Combinations of residues substituted with Cys to test for disulfide crosslinking and distances between C_β atoms predicted by models

SpoIVFA residue	BofA residue	C_β distance in previous model (Å)	C_β distance in AlphaFold model (Å)
H65	T65	29.7	23.8
S80	T65	6.4	15.0
S80	G69[a]	4.5	11.5
S80	A77	6.9	6.6
S80	A78	5.0	3.9
A81	V81	7.3	9.4
L83	A78	8.0	4.9
V84	T65	8.0	17.8
V84	A78	5.2	5.1
V84	V81	7.0	5.4
V84	I82	3.4	5.9
L85	F85	7.9	12.0
S87	I82	8.7	4.8
A88	I82	7.8	7.9
A88	I86	4.5	5.5

[a]Since G69 lacks a C_β atom, the distance to its C_α atom is reported.

Table S2. Confidence metrics for AlphaFold structural predictions of the SpoIVFB inhibition complex from various bacterial species

Species	Replicate	AlphaFold-3		AlphaFold-2 Multimer version 3		AlphaFold-2 Multimer version 2	
		ipTM	pTM	ipTM	pTM	ipTM	pTM
Bacillus subtilis	1	0.70	0.75	0.66	0.74	0.69	0.74
	2	0.70	0.75	0.66	0.74	0.69	0.74
	3	0.70	0.74	0.66	0.74	0.69	0.74
	4	0.70	0.75	0.66	0.74	0.69	0.74
	5	0.71	0.76	0.66	0.74	0.69	0.74
***Alkalihalobacillus halodurans* C-125**	1	0.73	0.78	0.71	0.77	x	x
	2	0.71	0.76	0.71	0.77	x	x
	3	0.71	0.75	0.71	0.77	x	x
	4	0.71	0.75	0.72	0.77	x	x
	5	0.71	0.76	0.71	0.77	x	x
***Paenibacillus polymyxa* SC2**	1	0.52	0.60	0.62	0.63	x	x
	2	0.45	0.54	0.63	0.64	x	x
	3	0.47	0.56	0.62	0.63	x	x
	4	0.49	0.59	0.62	0.63	x	x
	5	0.43	0.52	0.63	0.64	x	x
***Kyrpidia tusciae* DSM 2912**	1	0.45	0.53	0.57	0.64	x	x
	2	0.47	0.54	0.57	0.64	x	x
	3	0.45	0.53	0.57	0.64	x	x
	4	0.45	0.52	0.57	0.64	x	x
	5	0.45	0.54	0.57	0.64	x	x

Shown are the ipTM and pTM scores for the versions of AlphaFold shown at the top of the table. Each simulation was run with 5 independent replicates, that were each initiated using a different random seed. The reported ipTM and pTM scores are from the top-ranked model (out of 5) from each independent run.

Table S3. Plasmids used in this study

Name	Description	Construction	Reference
pAB5	Km^R; T7-Cys-less Pro-σ^K(1-127)-His_6/T7-Cys-less cytTM-SpoIVFB-$FLAG_2$-His_6/T7-Cys-less MBPΔ27BofA/single-Cys H65C SpoIVFA	pLK29 was subjected to SDM using primers JL-P19 and JL-P20, substituting H65C in SpoIVFA	This study
pAS10	Km^R; T7-Cys-less Pro-σ^K(1-127)-His_6/T7-Cys-less cytTM-SpoIVFB E44Q-$FLAG_2$-His_6/T7-Cys-less BofA/single-Cys V84C SpoIVFA	pSO242 was subjected to SDM using primers ES-P29 and ES-P30, substituting V84C in SpoIVFA	This study
pAS11	Km^R; T7-Cys-less Pro-σ^K(1-127)-His_6/T7-Cys-less cytTM-SpoIVFB E44Q-$FLAG_2$-His_6/T7-Cys-less BofA/single-Cys A88C SpoIVFA	pSO242 was subjected to SDM using primers ES-P35 and ES-P36, substituting A88C in SpoIVFA	This study
pAS12	Km^R; T7-Cys-less Pro-σ^K(1-127)-His_6/T7-Cys-less cytTM-SpoIVFB E44Q-$FLAG_2$-His_6/T7-Cys-less BofA T65S/single-Cys V84C SpoIVFA	pAS10 was subjected to SDM using primers LK-P3 and LK-P4, substituting T65S in BofA	This study
pAS13	Km^R; T7-Cys-less Pro-σ^K(1-127)-His_6/T7-Cys-less cytTM-SpoIVFB E44Q-$FLAG_2$-His_6/T7-single-Cys T65C BofA/single-Cys V84C SpoIVFA	pAS10 was subjected to SDM using primers ES-P43 and ES-P44, substituting T65C in BofA	This study
pAS14	Km^R; T7-Cys-less Pro-σ^K(1-127)-His_6/T7-Cys-less cytTM-SpoIVFB E44Q-$FLAG_2$-His_6/T7-single-Cys V81C BofA/single-Cys V84C SpoIVFA	pAS10 was subjected to SDM using primers ES-P51 and ES-P52, substituting V81C in BofA	This study
pAS15	Km^R; T7-Cys-less Pro-σ^K(1-127)-His_6/T7-Cys-less cytTM-SpoIVFB E44Q-$FLAG_2$-His_6/T7-Cys-less BofA V81S/single-Cys V84C SpoIVFA	pAS10 was subjected to SDM using primers LK-P5 and LK-P6, substituting V81S in BofA.	This study
pAS16	Km^R; T7-Cys-less Pro-σ^K(1-127)-His_6/T7-Cys-less cytTM-SpoIVFB E44Q-$FLAG_2$-His_6/T7-single-Cys I86C BofA/single-Cys A88C SpoIVFA	pAS11 was subjected to SDM using primers ES-P53 and ES-P54, substituting I86C in BofA.	This study
pAS17	Km^R; T7-Cys-less Pro-σ^K(1-127)-His6/T7-Cys-less cytTM-SpoIVFB E44Q-FLAG2-His6/T7-Cys-less BofA I86S/single-Cys A88C SpoIVFA	pAS11 was subjected to SDM using primers LK-P21 and LK-P22, substituting I86S in BofA	This study
pAS18	Km^R; T7-Cys-less Pro-σ^K(1-127)-His_6/T7-Cys-less cytTM-SpoIVFB-$FLAG_2$-His_6/T7-Cys-less MBPΔ27BofA T65S/Cys-less SpoIVFA	pLK29 was subjected to SDM using primers LK-P3 and LK-P4, substituting T65S in MBPΔ27BofA	This study
pAS19	Km^R; T7-Cys-less Pro-σ^K(1-127)-His_6/T7-Cys-less cytTM-SpoIVFB-$FLAG_2$-His_6/T7-single-Cys V81C MBPΔ27BofA/Cys-less SpoIVFA	pLK29 was subjected to SDM using primers ES-P51 and ES-P52, substituting V81C in MBPΔ27BofA	This study

pAS20	Km^R; T7-Cys-less Pro-σ^K(1-127)-His_6/T7-Cys-less cytTM-SpoIVFB-$FLAG_2$-His_6/T7-Cys-less MBPΔ27BofA V81S/Cys-less SpoIVFA	pLK29 was subjected to SDM using primers LK-P5 and LK-P6, substituting V81S in MBPΔ27BofA	This study
pAS21	Km^R; T7-Cys-less Pro-σ^K(1-127)-His_6/T7-Cys-less cytTM-SpoIVFB-$FLAG_2$-His_6/T7-Cys-less MBPΔ27BofA/single-Cys A88C SpoIVFA	pLK29 was subjected to SDM using primers ES-P35 and ES-P36, substituting A88C in SpoIVFA	This study
pAS22	Km^R; T7-Cys-less Pro-σ^K(1-127)-His_6/T7-Cys-less cytTM-SpoIVFB-$FLAG_2$-His_6/T7-Cys-less MBPΔ27BofA/Cys-less SpoIVFA A88S	pLK29 was subjected to SDM using primers LK-P23 and LK-P24, substituting A88S in SpoIVFA	This study
pAS23	Km^R; T7-Cys-less Pro-σ^K(1-127)-His_6/T7-Cys-less cytTM-SpoIVFB-$FLAG_2$-His_6/T7-Cys-less MBPΔ27BofA I86S/Cys-less SpoIVFA	pLK29 was subjected to SDM using primers LK-P21 and LK-P22, substituting I86S in MBPΔ27BofA	This study
pES22	Km^R; T7-Cys-less Pro-σ^K(1-127)-His_6/T7-Cys-less cytTM-SpoIVFB E44Q-$FLAG_2$-His_6/T7-Cys-less MBPΔ27BofA/single-Cys V84C SpoIVFA	pSO139 was subjected to SDM using primers ES-P29 and ES-P30, substituting V84C in SpoIVFA	This study
pES23	Km^R; T7-Cys-less Pro-σ^K(1-127)-His_6/T7-Cys-less cytTM-SpoIVFB E44Q-$FLAG_2$-His_6/T7-Cys-less MBPΔ27BofA/single-Cys S80C SpoIVFA	pSO139 was subjected to SDM using primers ES-P31 and ES-P32 substituting S80C in SpoIVFA	This study
pES24	Km^R; T7-Cys-less Pro-σ^K(1-127)-His_6/T7-Cys-less cytTM-SpoIVFB E44Q-$FLAG_2$-His_6/T7-single-Cys I82C MBPΔ27BofA/single-Cys S87C SpoIVFA	pMB9 was subjected to SDM using primers ES-P33 and ES-P34, substituting S87C in SpoIVFA	This study
pES25	Km^R; T7-Cys-less Pro-σ^K(1-127)-His_6/T7-Cys-less cytTM-SpoIVFB E44Q-$FLAG_2$-His_6/T7-single-Cys I82C MBPΔ27BofA/single-Cys A88C SpoIVFA	pMB9 was subjected to SDM using primers ES-P35 and ES-P36, substituting A88C in SpoIVFA	This study
pES26	Km^R; T7-Cys-less Pro-σ^K(1-127)-His_6/T7-Cys-less cytTM-SpoIVFB E44Q-$FLAG_2$-His_6/T7-single-Cys F85C MBPΔ27BofA/single-Cys L85C SpoIVFA	pMB11 was subjected to SDM using primers ES-P37 and ES-P38, substituting L85C in SpoIVFA	This study
pES27	Km^R; T7-Cys-less Pro-σ^K(1-127)-His_6/T7-Cys-less cytTM-SpoIVFB E44Q-$FLAG_2$-His_6/T7-single-Cys T65C MBPΔ27BofA/single-Cys V84C SpoIVFA	pES22 was subjected to SDM using primers ES-P43 and ES-P44, substituting T65C in MBPΔ27BofA	This study
pES28	Km^R; T7-Cys-less Pro-σ^K(1-127)-His_6/T7-Cys-less cytTM-SpoIVFB E44Q-$FLAG_2$-His_6/T7-single-Cys A78C MBPΔ27BofA/single-Cys V84C SpoIVFA	pES22 was subjected to SDM using primers ES-P49 and ES-p50, substituting A78C in MBPΔ27BofA	This study
pES29	Km^R; T7-Cys-less Pro-σ^K(1-127)-His_6/T7-Cys-less cytTM-SpoIVFB E44Q-$FLAG_2$-	pES22 was subjected to SDM using primers ES-P51 and ES-	This study

	His_6/T7-single-Cys V81C MBPΔ27BofA/single-Cys V84C SpoIVFA	P52, substituting V81C in MBPΔ27BofA	
pES30	Km^R; T7-Cys-less Pro-σ^K(1-127)-His_6/T7-Cys-less cytTM-SpoIVFB E44Q-$FLAG_2$-His_6/T7-single-Cys T65C MBPΔ27BofA/single-Cys S80C SpoIVFA	pES23 was subjected to SDM using primers ES-P43 and ES-P44, substituting T65C in MBPΔ27BofA	This study
pES31	Km^R; T7-Cys-less Pro-σ^K(1-127)-His_6/T7-Cys-less cytTM-SpoIVFB E44Q-$FLAG_2$-His_6/T7-single-Cys G69C MBPΔ27BofA/single-Cys S80C SpoIVFA	pES23 was subjected to SDM using primers ES-P45 and ES-P46, substituting G69C in MBPΔ27BofA	This study
pES32	Km^R; T7-Cys-less Pro-σ^K(1-127)-His_6/T7-Cys-less cytTM-SpoIVFB E44Q-$FLAG_2$-His_6/T7-single-Cys A77C MBPΔ27BofA/single-Cys S80C SpoIVFA	pES23 was subjected to SDM using primers ES-P47 and ES-P48, substituting A77C in MBPΔ27BofA	This study
pES33	Km^R; T7-Cys-less Pro-σ^K(1-127)-His_6/T7-Cys-less cytTM-SpoIVFB E44Q-$FLAG_2$-His_6/T7-single-Cys A78C MBPΔ27BofA/single-Cys S80C SpoIVFA	pES23 was subjected to SDM using primers ES-P49 and ES-P50, substituting A78C in MBPΔ27BofA	This study
pES34	Km^R; T7-Cys-less Pro-σ^K(1-127)-His_6/T7-Cys-less cytTM-SpoIVFB E44Q-$FLAG_2$-His_6/T7-single-Cys I82C MBPΔ27BofA/single-Cys V84C SpoIVFA	pMB9 was subjected to SDM using primers ES-P29 and ES-P30, substituting V84C in SpoIVFA	This study
pES35	Km^R; T7-Cys-less Pro-σ^K(1-127)-His_6/T7-Cys-less cytTM-SpoIVFB E44Q-$FLAG_2$-His_6/T7-single-Cys I86C MBPΔ27BofA/single-Cys A88C SpoIVFA	pES25 was subjected to SDM using primers ES-P53 and ES-P54, substituting C82I and I86C in MBPΔ27BofA	This study
pES36	Km^R; T7-Cys-less Pro-σ^K(1-127)-His_6/T7-Cys-less cytTM-SpoIVFB E44Q-$FLAG_2$-His_6/T7-single-Cys A78C MBPΔ27BofA/single-Cys L83C SpoIVFA	pES28 was subjected to SDM using primers ES-P39 and ES-P40, substituting C84V and L83C in SpoIVFA	This study
pES37	Km^R; T7-Cys-less Pro-σ^K(1-127)-His_6/T7-Cys-less cytTM-SpoIVFB E44Q-$FLAG_2$-His_6/T7-single-Cys V81C MBPΔ27BofA/single-Cys A81C SpoIVFA	pES29 was subjected to SDM using primers ES-P41 and ES-P42, substituting C84V and A81C in SpoIVFA	This study
pLK29	Km^R; T7-Cys-less Pro-σ^K(1-127)-His_6/T7-Cys-less cytTM-SpoIVFB-$FLAG_2$-His_6/T7-Cys-less MBPΔ27BofA/Cys-less SpoIVFA	pSO139 was subjected to SDM PCR using primers DP-P58 and DP-P59, substituting Q44E in SpoIVFB	This study
pLK40	Km^R; T7-Cys-less Pro-σ^K(1-127)-His_6/T7-Cys-less cytTM-SpoIVFB E44Q-$FLAG_2$-His_6/T7-Cys-less MBPΔ27BofA/single-Cys H65C SpoIVFA	pSO139 was subjected to SDM PCR using primers JL-P19 and JL-P20, substituting H65C in SpoIVFA	This study
pLK41	Km^R; T7-Cys-less Pro-σ^K(1-127)-His_6/T7-Cys-less cytTM-SpoIVFB E44Q-$FLAG_2$-His_6/T7-Cys-less MBPΔ27BofA I86S/single-Cys A88C SpoIVFA	pES35 was subjected to SDM using primers LK-P21 and LK-P22, substituting I86S in MBPΔ27BofA	This study

pLK42	Km^R; T7-Cys-less Pro-σ^K(1-127)-His_6/T7-Cys-less cytTM-SpoIVFB E44Q-$FLAG_2$-His_6/T7-Cys-less MBPΔ27BofA T65A/single-Cys V84C SpoIVFA	pES22 was subjected to SDM using primers LK-P25 and LK-P26, substituting T65A in MBPΔ27BofA	This study
pLK43	Km^R; T7-Cys-less Pro-σ^K(1-127)-His_6/T7-Cys-less cytTM-SpoIVFB E44Q-$FLAG_2$-His_6/T7-single-Cys I86C MBPΔ27BofA/Cys-less SpoIVFA A88S	pES35 was subjected to SDM using primers LK-P23 and LK-P24, substituting A88S in SpoIVFA	This study
pLK49	Km^R; T7-Cys-less Pro-σ^K(1-127)-His_6/T7-Cys-less cytTM-SpoIVFB E44Q-$FLAG_2$-His_6/T7-single-Cys T65C MBPΔ27BofA/single-Cys H65C SpoIVFA	pLK40 was subjected to SDM using primers ES-P43 and ES-P44, substituting T65C in MBPΔ27BofA	This study
pLK53	Km^R; T7-Cys-less Pro-σ^K(1-127)-His_6/T7-Cys-less cytTM-SpoIVFB-$FLAG_2$-His_6/T7-single-Cys I86C MBPΔ27BofA/Cys-less SpoIVFA	pLK29 was subjected to SDM using primers ES-P53 and ES-P54, substituting I86C in MBPΔ27BofA	This study
pLK56	Km^R; T7-Cys-less Pro-σ^K(1-127)-His_6/T7-Cys-less cytTM-SpoIVFB-$FLAG_2$-His_6/T7-Cys-less MBPΔ27BofA/Cys-less SpoIVFA V84S	pLK29 was subjected to SDM using primers LK-P1 and LK-P2, substituting V84S in SpoIVFA	This study
pLK57	Km^R; T7-Cys-less Pro-σ^K(1-127)-His_6/T7-Cys-less cytTM-SpoIVFB-$FLAG_2$-His_6/T7-Cys-less MBPΔ27BofA/single-Cys V84C SpoIVFA	pLK29 was subjected to SDM using primers ES-P29 and ES-P30, substituting V84C in SpoIVFA	This study
pLK58	Km^R; T7-Cys-less Pro-σ^K(1-127)-His_6/T7-Cys-less cytTM-SpoIVFB-$FLAG_2$-His_6/T7-single-Cys T65C MBPΔ27BofA/Cys-less SpoIVFA	pLK29 was subjected to SDM using primers ES-P43 and ES-P44, substituting T65C in MBPΔ27BofA	This study
pLK59	Km^R; T7-Cys-less Pro-σ^K(1-127)-His_6/T7-Cys-less cytTM-SpoIVFB-$FLAG_2$-His_6/T7-MBPΔ27BofA N48A/Cys-less SpoIVFA	pLK29 was subjected to SDM using primers SO-P52 and SO-P53, substituting S46C N48A in MBPΔ27BofA	This study
pMB9	Km^R; T7-Cys-less Pro-σ^K(1-127)-His_6/T7-Cys-less cytTM-SpoIVFB E44Q-$FLAG_2$-His_6/T7-single-Cys I82C MBPΔ27BofA/Cys-less SpoIVFA	pSO139 was subjected to SDM using primers MB-P1 and MB-P2, substituting I82C in MBPΔ27BofA	This study
pMB11	Km^R; T7-Cys-less Pro-σ^K(1-127)-His_6/T7-Cys-less cytTM-SpoIVFB E44Q-$FLAG_2$-His_6/T7-single-Cys F85C MBPΔ27BofA/Cys-less SpoIVFA	pSO139 was subjected to SDM using primers MB-P5 and MB-P6, substituting F85C in MBPΔ27BofA	This study
pSO139	Km^R; T7-Cys-less Pro-σ^K(1-127)-His_6/T7-Cys-less cytTM-SpoIVFB E44Q-$FLAG_2$-His_6/T7-Cys-less MBPΔ27BofA/Cys-less SpoIVFA		(1)

Table S4. Primers used in this study

Primer	Sequence[a]
DP-P58	CTGATTGTATTGATTCAT**GAG**CTGGGGCATGCTGCTCTG
DP-P59	CAGAGCAGCATGCCCCAG**CTC**ATGAATCAATACAATCAG
ES-P29	CTTCTGTCGGCCTTACTT**TG**TCTCGTTTCAGCTATAGC
ES-P30	GCTATAGCTGAAACGAGA**CA**AAGTAAGGCCGACAGAAG
ES-P31	CAATTATCCTGAAATTACTTCTGT**GT**GCCTTACTTGTTCTCGTTTC
ES-P32	GAAACGAGAACAAGTAAGGC**AC**ACAGAAGTAATTTCAGGATAATTG
ES-P33	CCTTACTTGTTCTCGTTT**GT**GCTATAGCCTATAAAACAAAC
ES-P34	GTTTGTTTTATAGGCTATAGC**AC**AAACGAGAACAAGTAAGG
ES-P35	CTTACTTGTTCTCGTTTCA**TG**TATAGCCTATAAAACAAAC
ES-P36	GTTTGTTTTATAGGCTATA**CA**TGAAACGAGAACAAGTAAG
ES-P37	CTGTCGGCCTTACTTGTT**TG**CGTTTCAGCTATAGCCTATAAAAC
ES-P38	GTTTTATAGGCTATAGCTGAAACG**CA**AACAAGTAAGGCCGACAG
ES-P39	GAAATTACTTCTGTCGGCCTTA**TG**T**GT**TCTCGTTTCAGCTATAGC
ES-P40	GCTATAGCTGAAACGAGA**AC**A**CA**TAAGGCCGACAGAAGTAATTTC
ES-P41	CTGAAATTACTTCTGTCG**TG**CTTACTT**GT**TCTCGTTTCAGCTATAGC
ES-P42	GCTATAGCTGAAACGAGA**AC**AAGTAAG**CA**CGACAGAAGTAATTTCAG
ES-P43	CCGATTAATCTGGTTACA**TGT**GCTATCAGCGGAATTTTAG
ES-P44	CTAAAATTCCGCTGATAGC**ACA**TGTAACCAGATTAATCGG
ES-P45	GTTACAACAGCTATCAGC**T**G**T**ATTTTAGGAATACCCGGAATAG
ES-P46	CTATTCCGGGTATTCCTAAAAT**A**C**A**GCTGATAGCTGTTGTAAC
ES-P47	GAATTTTAGGAATACCCGGAATA**TG**TGCGTTAGTCGTCATTAAG
ES-P48	CTTAATGACGACTAACGCA**CA**TATTCCGGGTATTCCTAAAATTC
ES-P49	GGAATACCCGGAATAGCT**TGT**TTAGTCGTCATTAAGCAATTTATC
ES-P50	GATAAATTGCTTAATGACGACTAA**ACA**AGCTATTCCGGGTATTCC
ES-P51	GGAATAGCTGCGTTAGTC**TG**CATTAAGCAATTTATCATTTAAG
ES-P52	CTTAAATGATAAATTGCTTAATG**CA**GACTAACGCAGCTATTCC
ES-P53	GAATAGCTGCGTTAGTCGTC**AT**TAAGCAATTT**TG**CATTTAAGGATCCGAAGG
ES-P54	CCTTCGGATCCTTAAATG**CA**AAATTGCTTA**AT**GACGACTAACGCAGCTATTC
JL-P19	CCAACATTCAACGGAAAA**TGC**CCATTGGTGAAAACAGATTC
JL-P20	ATCTGTTTTCACCAATGG**GCA**TTTTCCGTTGAATGTTGG
LK-P1	CTTCTGTCGGCCTTACTT**TCT**CTCGTTTCAGCTATAGC
LK-P2	GCTATAGCTGAAACGAG**AGA**AAGTAAGGCCGACAGAAG
LK-P3	CCGATTAATCTGGTTACA**TCT**GCTATCAGCGGAATTTTAG
LK-P4	CTAAAATTCCGCTGATAGC**AGA**TGTAACCAGATTAATCGG
LK-P5	GGAATAGCTGCGTTAGTC**AGC**ATTAAGCAATTTATCATTTAAG
LK-P6	CTTAAATGATAAATTGCTTAAT**GCT**GACTAACGCAGCTATTCC
LK-P21	GTCGTCATTAAGCAATTT**AGC**ATTTAAGGATCCGAAGG
LK-P22	CCTTCGGATCCTTAAAT**GCT**AAATTGCTTAATGACGAC
LK-P23	CTTACTTGTTCTCGTTTCA**TCT**ATAGCCTATAAAACAAAC
LK-P24	GTTTGTTTTATAGGCTAT**AGA**TGAAACGAGAACAAGTAAG
LK-P25	CCGATTAATCTGGTTACA**GCA**GCTATCAGCGGAATTTTAG

LK-P26	CTAAAATTCCGCTGATAGC**TGC**TGTAACCAGATTAATCGG
MB-P1	GAATAGCTGCGTTAGTCGTC**TGT**AAGCAATTTATCATTTAA
MB-P2	TTAAATGATAAATTGCTT**ACA**GACGACTAACGCAGCTATTC
MB-P5	GCGTTAGTCGTCATTAAGCAA**TGT**ATCATTTAAGGATCCGAAG
MB-P6	CTTCGGATCCTTAAATGAT**ACA**TTGCTTAATGACGACTAACGC
SO-P52	TTGCTGCTGGTT**T**GTGTA**GC**TATGTTTGGCGGCAGTCT
SO-P53	AGACTGCCGCCAAACATA**GC**TACAC**A**AACCAGCAGCAA

[a]Mutagenic nucleotides are shown in bold.

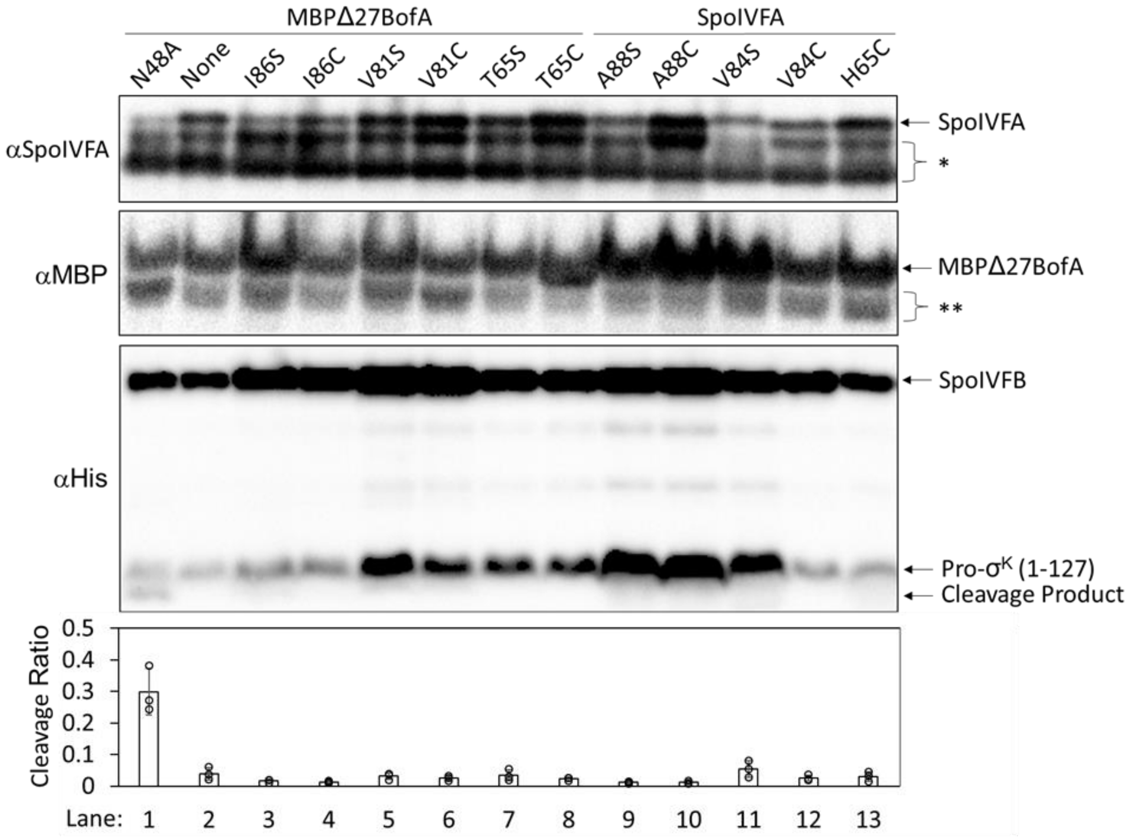

Fig. S1 Effects of cysteine or serine substitutions in MBPΔ27BofA or SpoIVFA on inhibition of Pro-σ^K(1-127) cleavage in *E. coli*. pET Quartet plasmids were used to produce Pro-σ^K(1-127), catalytically active SpoIVFB, SpoIVFA, and MBPΔ27BofA (lane 2) or derivatives of with the indicated substitution. The derivative with the N48A substitution in MBPΔ27BofA served as a control to show partial loss of cleavage inhibition (1). Samples collected after 2 hr of IPTG induction were subjected to immunoblot analysis with antibodies against SpoIVFA (top), MBP (middle), and penta-His (bottom). The single asterisk (*) indicates cross-reacting proteins below SpoIVFA and the double asterisk (**) indicates breakdown species of MBPΔ27BofA. The graph shows quantification of the cleavage ratio (cleavage product/[Pro-σ^K(1–127)+cleavage product]) for three biological replicates (open circles). Bars show the average and error bars show 1 standard deviation.

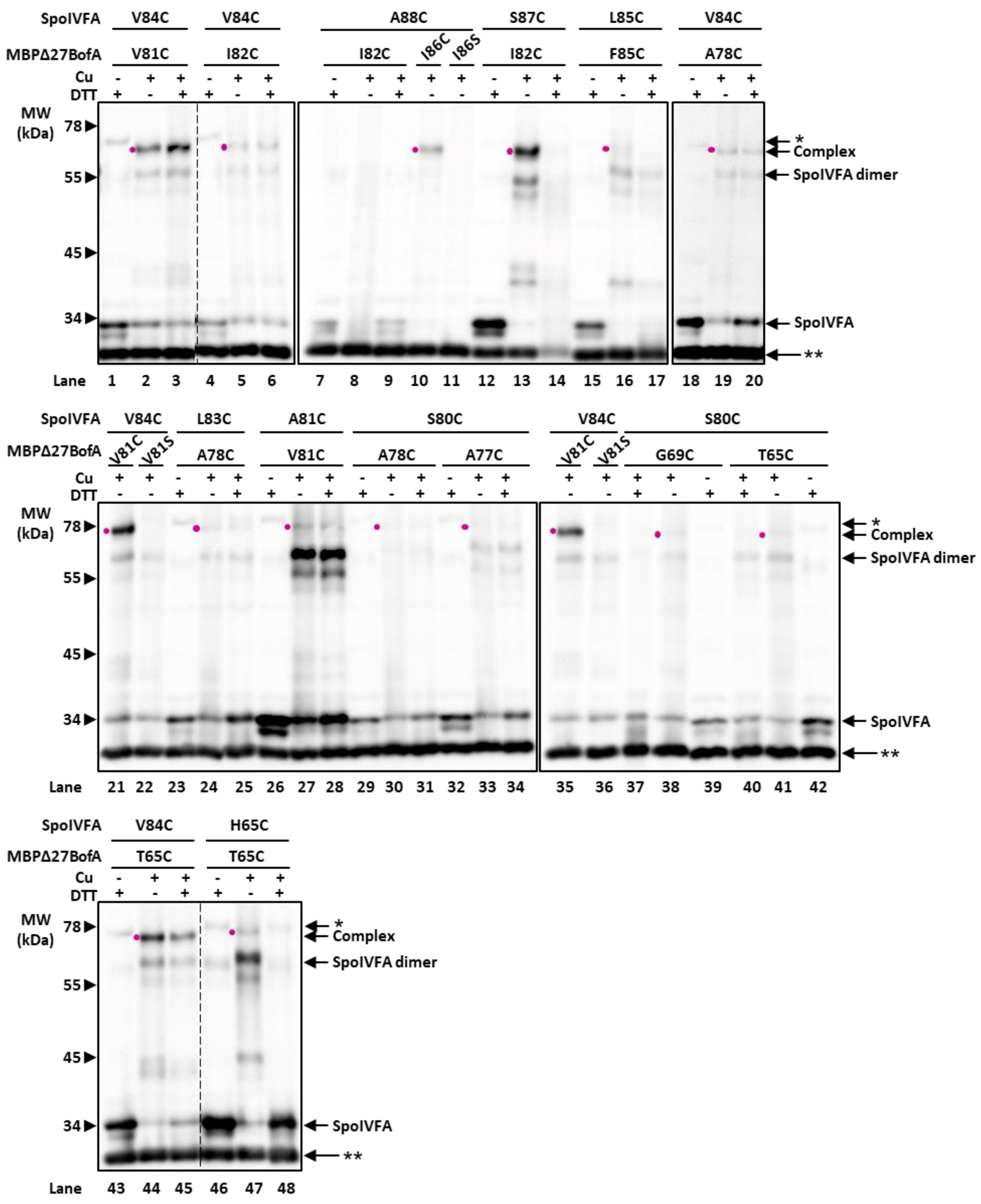

Fig. S2 Disulfide crosslinking of combinations of SpoIVFA and MBPΔ27BofA variants. Anti-SpoIVFA immunoblots of crosslinking performed as described in the Figure 3 legend. Species migrating at positions expected for SpoIVFA, SpoIVFA dimer, and complex between SpoIVFA and MBPΔ27BofA are indicated. The complex, if present, is also identified with a dot (•) next to the sample treated only with Cu. A star (*) denotes an unidentified species migrating slower than complex and most obvious in samples treated only with DTT. A double star (**) indicates a cross-reacting protein. The position of migration of protein molecular weight (MW) markers is shown on the blot at left. Representative results from at least two biological replicates are shown.

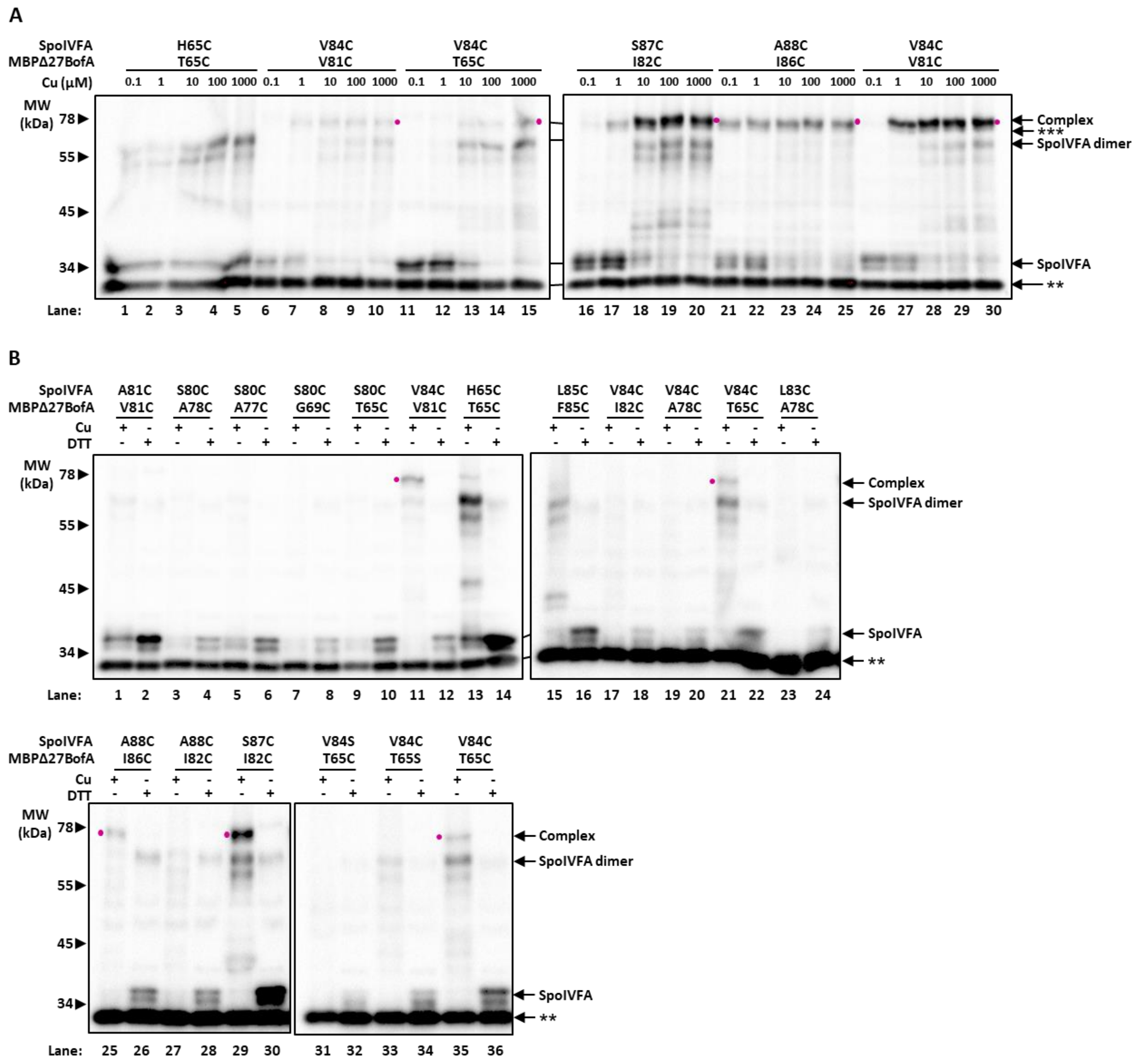

Fig. S3 Biological replicates of disulfide crosslinking at different oxidant concentrations and under stringent conditions. A) Biological replicate of the experiment shown in Figure 5. The results show that the relative ability of crosslinks to form at different oxidant concentrations is reproducible. B) Biological replicate of the experiment shown in Figure 6. The results show that the ability of crosslinks to form under stringent conditions is reproducible.

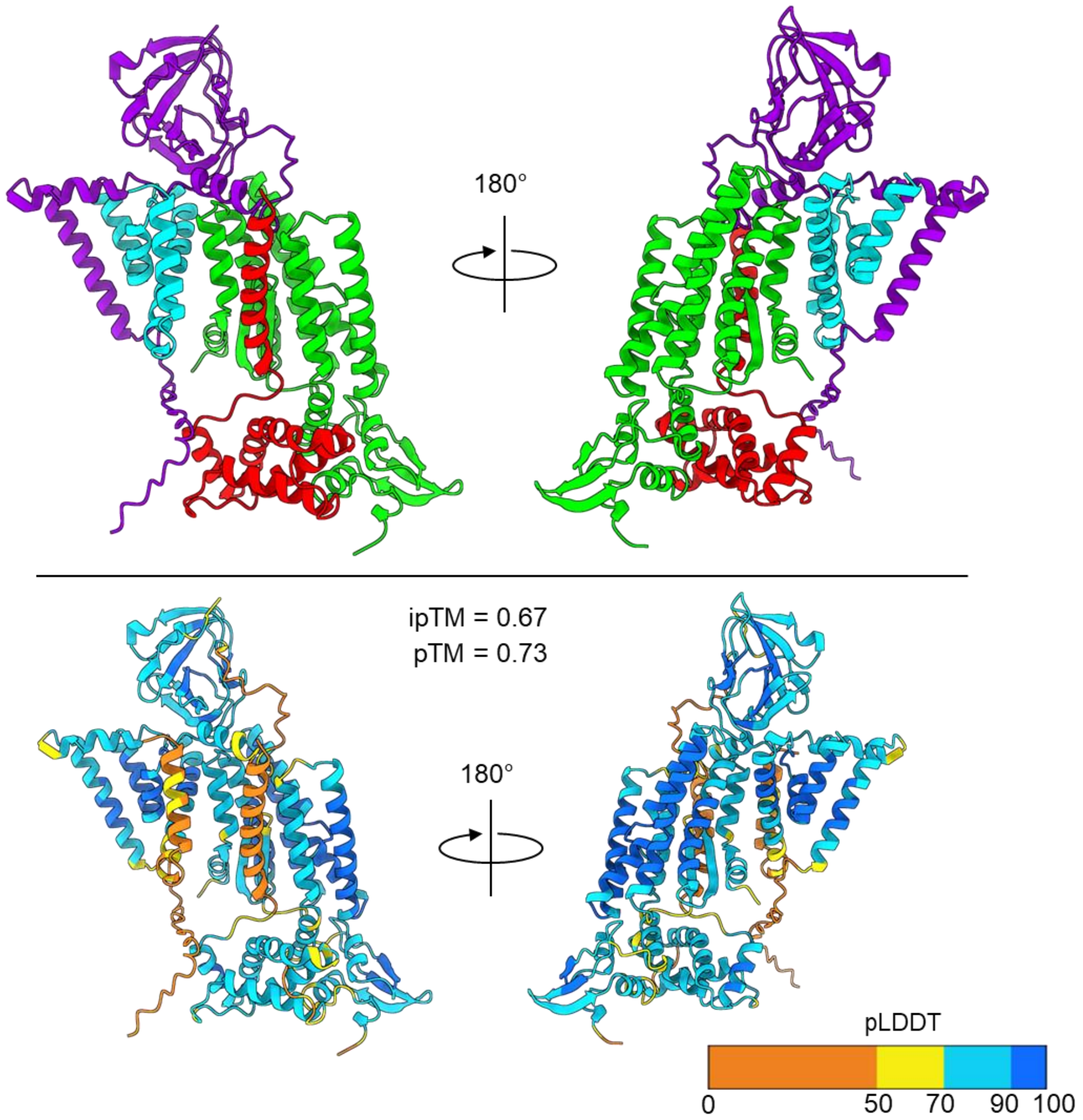

Fig. S4 AlphaFold-2 Multimer version 2 structural prediction of the SpoIVFB inhibition complex. In the top part, two rotated views of the complex are shown with SpoIVFB colored green, BofA cyan, Pro-σ^K(1-127) red, and SpoIVFA purple. The bottom part shows the complexes in the same orientation as the top part, but colored according to pLDDT score. The overall prediction ipTM and pTM scores are shown above the models.

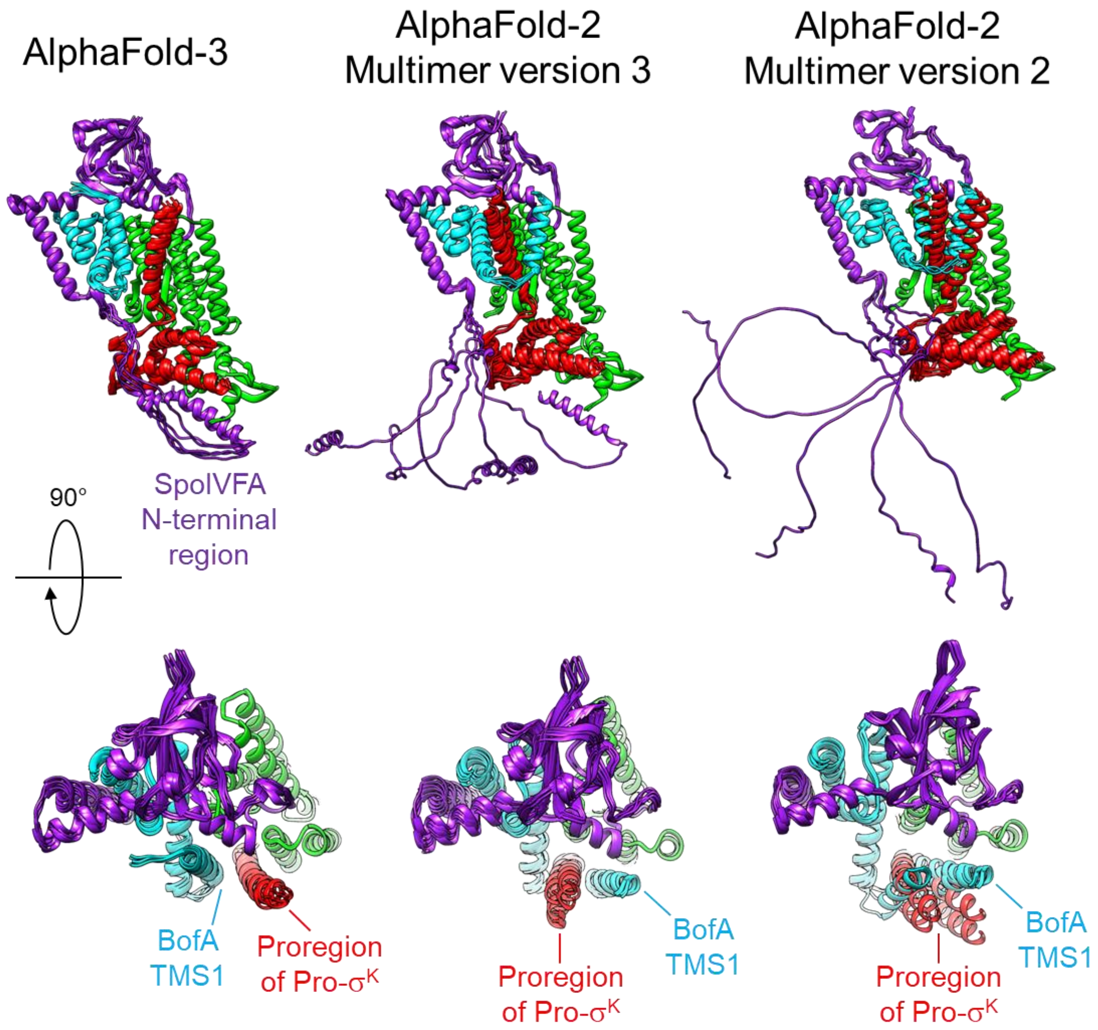

Fig. S5 Structural predictions of the SpoIVFB inhibition complex with different versions of AlphaFold. The top-ranked models from five independent runs of each software version are overlaid upon one another, with alignment of all models based on the SpoIVFB component. SpoIVFB is colored green, BofA is shown in cyan, Pro-σ^K(1-127) is shown in red, and SpoIVFA is shown in purple. Predictions using AlphaFold-3 versus AlphaFold-2 Multimer show very similar predictions except in two key locations, which are labeled. First, AlphaFold-3 is more confident in modeling of the SpoIVFA N-terminal region, which is more disordered in predictions from the AlphaFold-2 Multimer versions. Second, the relative configurations of the Proregion of Pro-σ^K(1-127) and BofA transmembrane segment 1 (TMS1) differ depending on which version of AlphaFold was utilized.

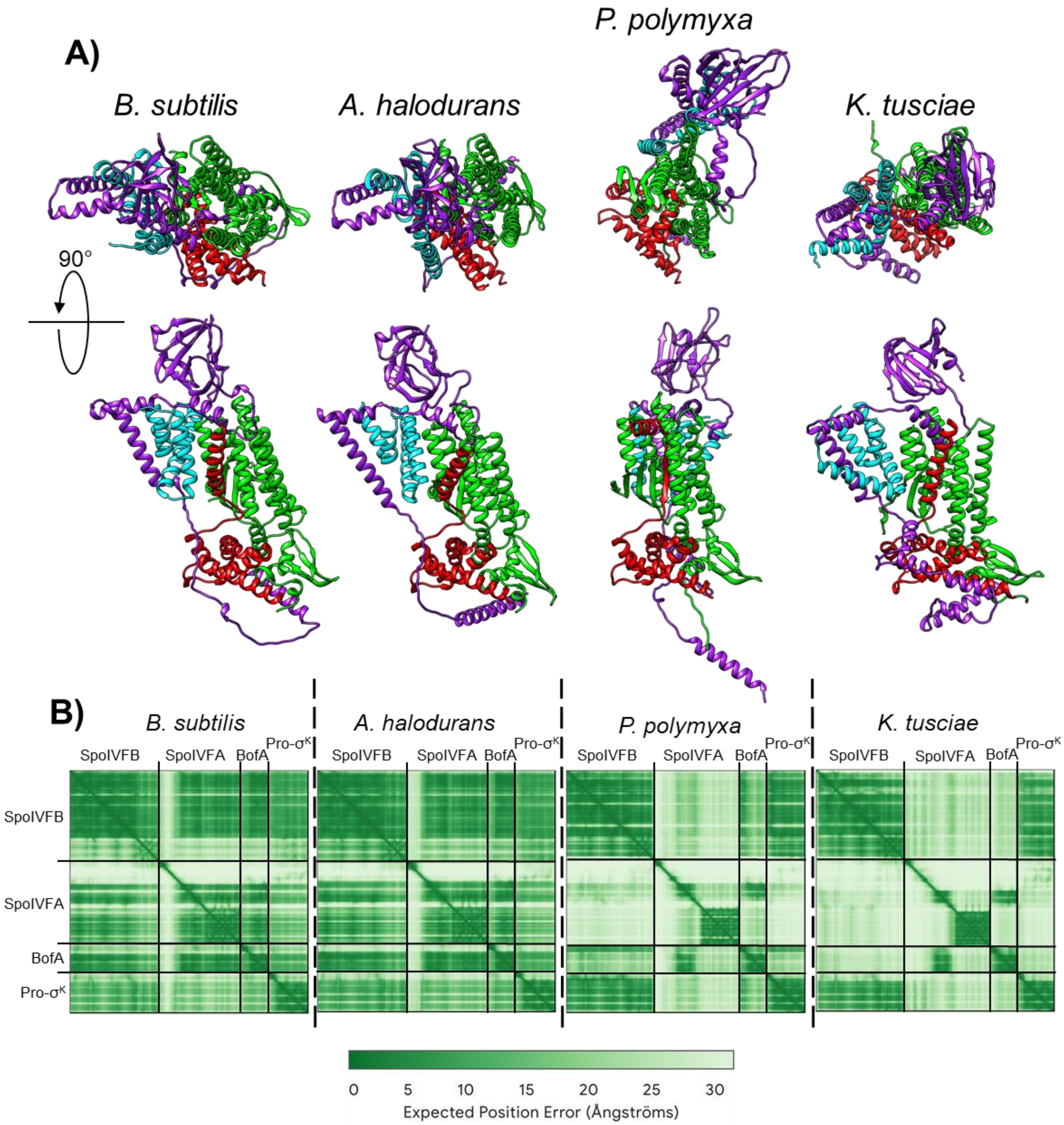

Fig. S6 Structural predictions of the SpoIVFB inhibition complex from *B. subtilis* and three other bacterial species. A) The top-ranked model for each complex from an AlphaFold-3 prediction is shown. All models are shown with the same relative orientation of the SpoIVFB component. SpoIVFB is colored green, BofA is shown in cyan, Pro-σ^K(1-127) is shown in red, and SpoIVFA is shown in purple. B) Predicted Aligned Error (PAE) plots are shown for the four predicted complexes. Black vertical and horizontal lines indicate the boundaries of individual protein chains.

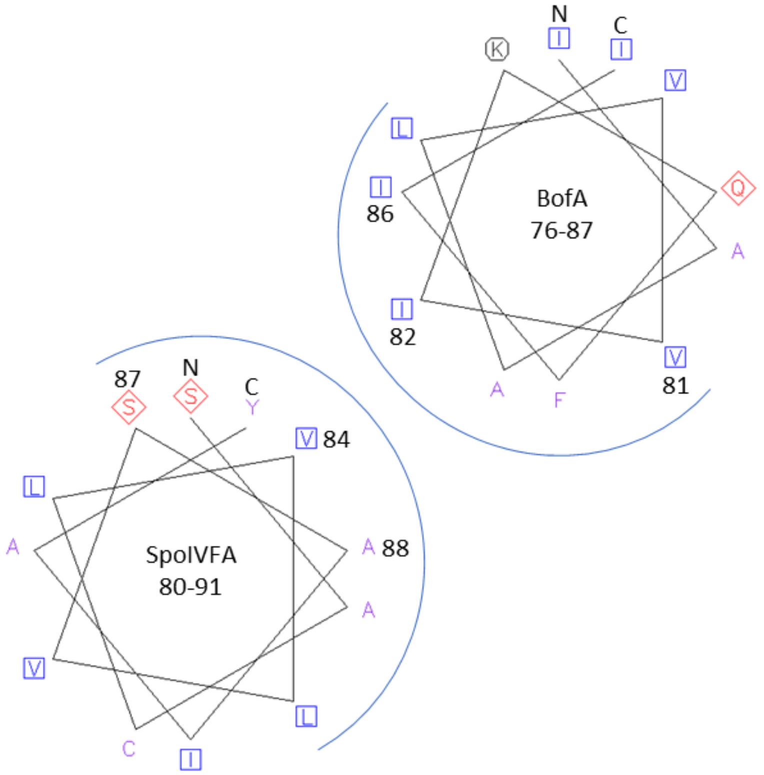

Fig. S7 Hydrophobic faces of a short α-helix at the BofA C-terminal tip and the C-terminal part of the SpoIVFA transmembrane segment likely interact. Helical wheel projections of BofA residues 76-87 and SpoIVFA residues 80-91 are arranged to reflect their interacting faces (arcs) based on our disulfide crosslinking results (numbered residues) and models shown in Figures 4B and 7B. The N- and C-terminal ends of each helix are indicated (N and C, respectively) and extend one to five residues beyond the residues shown to disulfide crosslink when Cys-subtituted. SpoIVFA S87 is polar and hence an exception to the otherwise hydrophobic faces likely to interact.

References

1. Olenic S, Heo L, Feig M, Kroos L. 2022. Inhibitory proteins block substrate access by occupying the active site cleft of *Bacillus subtilis* intramembrane protease SpoIVFB. eLife 11:e74275.
